# Cryptocurrencies and future financial crime

**DOI:** 10.1186/s40163-021-00163-8

**Published:** 2022-01-05

**Authors:** Arianna Trozze, Josh Kamps, Eray Arda Akartuna, Florian J. Hetzel, Bennett Kleinberg, Toby Davies, Shane D. Johnson

**Affiliations:** 1 Dawes Centre for Future Crime, University College London, 35 Tavistock Square, London, WC1H 9EZ UK; 2 Department of Security and Crime Science, University College London, 35 Tavistock Square, London, WC1H 9EZ UK; 3 Department of Methodology & Statistics, Tilburg University, Tilburg, The Netherlands; 4 Department of Computer Science, University College London, Gower Street, London, WC1E 6EA UK

**Keywords:** Cryptocurrencies, Fraud, Ponzi Schemes, Bitcoin, Virtual Currency

## Abstract

**Background:**

Cryptocurrency fraud has become a growing global concern, with various governments reporting an increase in the frequency of and losses from cryptocurrency scams. Despite increasing fraudulent activity involving cryptocurrencies, research on the potential of cryptocurrencies for fraud has not been examined in a systematic study. This review examines the current state of knowledge about what kinds of cryptocurrency fraud currently exist, or are expected to exist in the future, and provides comprehensive definitions of the frauds identified.

**Methods:**

The study involved a scoping review of academic research and grey literature on cryptocurrency fraud and a 1.5-day expert consensus exercise. The review followed the PRISMA-ScR protocol, with eligibility criteria based on language, publication type, relevance to cryptocurrency fraud, and evidence provided. Researchers screened 391 academic records, 106 of which went on to the eligibility phase, and 63 of which were ultimately analysed. We screened 394 grey literature sources, 128 of which passed on to the eligibility phase, and 53 of which were included in our review. The expert consensus exercise was attended by high-profile participants from the private sector, government, and academia. It involved problem planning and analysis activities and discussion about the future of cryptocurrency crime.

**Results:**

The academic literature identified 29 different types of cryptocurrency fraud; the grey literature discussed 32 types, 14 of which were not identified in the academic literature (i.e., 47 unique types in total). Ponzi schemes and (synonymous) high yield investment programmes were most discussed across all literature. Participants in the expert consensus exercise ranked pump-and-dump schemes and ransomware as the most profitable and feasible threats, though pump-and-dumps were, notably, perceived as the least harmful type of fraud.

**Conclusions:**

The findings of this scoping review suggest cryptocurrency fraud research is rapidly developing in volume and breadth, though we remain at an early stage of thinking about future problems and scenarios involving cryptocurrencies. The findings of this work emphasise the need for better collaboration across sectors and consensus on definitions surrounding cryptocurrency fraud to address the problems identified.

## Background

Cryptocurrency fraud has become a growing concern worldwide. Between 2017 and 2018, the Australian Competition and Consumer Commission (2019) registered a 190% increase in losses for victims of scams involving cryptocurrencies. In 2019, the United Kingdom Financial Conduct Authority issued a warning to the public after cryptocurrency scam reports tripled (Financial Conduct Authority, 2019). This trajectory of criminals defrauding individuals who have purchased or transacted using cryptocurrencies (cryptocurrency ‘users’) suggests the cryptocurrency space offers yet unexploited opportunities for crime.

The rapid surge in defrauded cryptocurrency users appears to have outpaced corresponding research efforts. Yli-Huumo, et al. (2016) conducted a literature review to identify key blockchain research areas. Fourteen out of 41 reviewed papers addressed Bitcoin blockchain security challenges. However, only one publication examined fraud associated with blockchain ecosystems (Vasek & Moore, 2015). This points to a lack of research investigating deception and misrepresentation for financial gain as a challenge for cryptocurrencies, and the forms of fraud that might occur. The aim of this paper is to understand which types of cryptocurrency fraud have thus far been identified, which types might develop in the future, and how these threats are perceived by researchers and other stakeholders. To this end, we present findings from two complementary studies: a scoping review of the state of published knowledge relating to cryptocurrency fraud, and an expert consensus exercise involving participants from various stakeholder organisations.

### A primer on cryptocurrencies

In this section, we provide a brief overview of the key principles of cryptocurrencies—with a focus on Bitcoin in particular—to provide context for the discussion of fraudulent exploitation that follows. While this outline is high-level, the interested reader is referred to both the original Bitcoin whitepaper (Nakamoto, 2008) or the textbook by Narayanan et al. (2016) for further details.

In 2008, an individual or group under the pseudonym Satoshi Nakamoto published a whitepaper entitled, ‘Bitcoin: A Peer-to-Peer Electronic Cash System’ (Nakamoto, 2008). This paper discussed a system through which parties could transact directly, without intermediary financial institutions. Bitcoin would rely on cryptography rather than central banks, law enforcement, and anti-counterfeiting measures to ensure security (Narayanan et al., 2016). Bitcoin’s market capitalisation has grown significantly since its implementation in 2009, and currently stands at $668 billion (CoinMarketCap, 2021). Bitcoin’s creation has sparked thousands of other cryptocurrencies which share similar tenets and technology; the total cryptocurrency market capitalisation is $1.6 trillion (CoinMarketCap, 2021).

Bitcoin and other cryptocurrencies share three common principles: *decentralisation*, *pseudo-anonymity*, and *transparency*. They are *decentralised* in that, rather than being governed by any single institution, they are administered via a peer-to-peer network, the majority of which must agree on which transactions and branch of a distributed digital ledger (the ‘blockchain’) are valid. They are *pseudo-anonymous* because, instead of usernames or account numbers, Bitcoin uses hashes of public keys to identify users, forming a system of ‘decentralised identity management’ decoupled from real-world identities. Cryptocurrencies are considered only pseudo-anonymous (rather than fully anonymous) due to the transparent nature of their transactions, despite not being explicitly connected with particular individuals and companies (Meiklejohn et al., 2016). *Transparency* results from the fact that all transactions that have ever occurred are recorded on the publicly available blockchain.

When someone creates a transaction, it is broadcast to all the peers in the network. To create a transaction, the user must have a pair of alphanumeric digital keys, comprising a public key (the hash of which identifies the user, and is analogous to an account address) and a private key (analogous to a PIN). Participants use their keys for digital signatures, to prove that they own the Bitcoin they are sending, and to specify the new owner.

The ‘miners’, a specialised subset of peers, collect contemporaneous transactions into a ‘block’ (one element of the ‘blockchain’). They compete to find a correct answer to a computationally hard puzzle—finding an input to a hash function which produces a particular output. Once one of the miners, after attempting many random inputs, finds a correct one, they broadcast the block to the network. This is referred to as Proof of Work (‘PoW’) because the nature of the puzzle means that, to find the correct input, the miner must have expended significant computational resources.^1^

Miners are rewarded for their work—at the time of writing, the reward for finding a correct block is 6.25 Bitcoin (Conway, 2021). These 6.25 Bitcoin are created and enter circulation once the miner finds a block, through what is called a ‘coinbase’ transaction (until the maximum amount of Bitcoin, as specified in Nakamoto’s paper—21 million—are minted). The reward is halved approximately every 4 years.

After a candidate block has been broadcast, a consensus process begins to establish whether the block is valid and should be added to the main ledger. Other miners perform a computational test on the transactions within the block and the PoW from the original miner: if this test gives the correct output, the block is considered valid.

They then add the next blocks to whichever chain they think is the correct one. At any given time, there may be multiple branches of the blockchain, but generally the longest one is the most valid. Importantly, participants can only add blocks to the blockchain and are unable to change previous blocks. Once a transaction is executed, it is irreversible. This consensus mechanism prevents what is known as ‘double-spending’, whereby a user could attempt to spend the same Bitcoin again. Consensus on the most valid chain requires agreement of at least 51% of miners and is usually achieved after about six blocks (Narayanan et al., 2016). An attack in which a miner or group of miners attempts to manipulate this by controlling 51% of the hashing power—requiring tremendous computational resources—is referred to as a ‘51% attack’.

There are a variety of ways users can store their cryptocurrencies, which effectively means storing their private key. Storage can be either ‘hot’ (online) or ‘cold’ (offline). Offline storage may involve a physical wallet locked in a safe or a key stored locally in a file on one’s computer. Though cold storage is generally safer, if one loses his/her private key or it is stolen, the coins are lost forever. Online wallets are often hosted through custodial wallet provider services, which manage users’ private keys; in exchange, the user sacrifices some anonymity, security, and control. Cryptocurrency exchanges are another type of online service and enable users to convert between fiat currencies backed by governments and cryptocurrencies and among different cryptocurrencies. Many offer custodial online wallets.

While Bitcoin was the first cryptocurrency, and is the prototypical example of the concept, a range of alternative coins and services have subsequently been created for cryptocurrency users who desire more anonymity. For example, Monero obscures wallet addresses and transactions (Keller et al., 2021). Individuals may also use ‘mixers’ or ‘tumblers’ to further obfuscate the origin of their funds. (Möser et al., 2013).

Another particularly prominent project in the field is Ethereum, which is a distributed virtual machine. Ethereum accounts enable smart contracts, which are computer programmes that automatically execute contracts, in the form of if-else statements (e.g., if a product is received, then release the funds) (Narayanan et al., 2016). The smart contract code is publicly visible on the blockchain and immutable. Smart contracts allow parties to enter contracts without needing to trust one another, or a third party, for execution. Rather, the parties can be confident that the contract will be carried out as agreed, so long as they trust its code (Bartoletti et al., 2020).

### Research approach

To determine the state of knowledge on which types of cryptocurrency fraud currently exist or will exist in the future, as well as the defining characteristics of these frauds, we conducted a scoping study in three steps. The first was a scoping review of published academic research on cryptocurrency fraud. This was followed by a 1.5-day in-person consensus exercise to elicit expert opinion on current and future threats, and to identify priorities for future work. The final step involved an updated search of the academic literature and a review of the grey literature.

## Scoping review

Scoping reviews are a replicable method of knowledge synthesis when it is unclear what has been already published on a given topic (Arksey & O’Malley, 2005; Levac et al., 2010; Munn et al., 2018; Paré et al., 2015; Peters et al., 2015; Peterson et al., 2017; Pham et al., 2014). The objective of this scoping review was to describe current research into cryptocurrency fraud. For the purpose of this review, we consider a cryptocurrency to be any electronic payment system which uses cryptography to secure peer-to-peer transactions (Nakamoto, 2008). Moreover, we define fraud as misrepresentation to gain some (financial) advantage (Law & Martin, 2009).

### Methods

#### Protocol

This review followed the *Preferred Reporting Items for Systematic reviews and Meta-Analyses extension for Scoping Reviews* (PRISMA-ScR) protocol (Moher et al., 2009).

#### Eligibility criteria

To be considered for this scoping review, published studies had to meet various eligibility criteria. First, we limited our review to publications written in English as we relied entirely on our reviewers’ language skills. The academic literature portion of the scoping review exclusively focused on academic articles such as peer-reviewed journals and conference papers due to the study’s aim of mapping out current research activities. The grey literature review included reports, publications, and alerts. By implication, the review excludes publications such as blog posts, op-eds, presentations, newsletters, marketing materials, correspondence, and magazine or newspaper articles.

Second, studies eligible for this review had to address cryptocurrency fraud in some form. As a minimum, a publication had to discuss at least one scam type related to cryptocurrencies. However, it was not necessary to dedicate an entire publication to this topic. Additionally, publications from the grey literature needed to be authored by a governmental organisation or a private sector company—publications from non-governmental or religious organisations were excluded.

Finally, statements about frauds exploiting cryptocurrency environments had to be based on empirical evidence. Studies had to report at least anecdotal evidence of the scams. If a study did not meet one of the eligibility criteria, we excluded it.

The review used Google Scholar (GS) to identify academic studies for review and Google’s Search Engine to identify private and public sector publications potentially eligible for review.^2^ One of the authors (AT) performed the final and most recent search on GS and Google’s Search Engine in November 2020.

#### Search strategy

Table 1 shows the search strings used. We split the search string into two separate queries because GS restricted searches to 256 characters.^3^ Moreover, we used inverted commas to limit the search to exact key phrases to avoid retrieving too many irrelevant records. The searches included academic and legal articles but excluded patents and citations. Searches were not limited to a given period; most publications were released in the last decade owing to the recency of the topic.

**Table 1:** Queries for the literature selection in Google Scholar

Label	Search string
Query 1	"cryptocurrency fraud" OR "cryptocurrency scam" OR "virtual currency fraud" OR "virtual currency scam" OR "digital currency fraud" OR "digital currency scam"
Query 2	"cryptocurrency frauds" OR "cryptocurrency scams" OR "virtual currency frauds" OR "virtual currency scams" OR "digital currency frauds" OR "digital currency scams"

We used the same two search strings to identify grey literature publications, with the addition of the following parameters: the file type should be a PDF and the text should be in English.

#### Selection of sources of evidence

Two reviewers (EA and FH) separately selected the publications eligible for the scoping review in two steps. First, each reviewer independently screened the title and abstract of the publications for language, publication type, and relevance to cryptocurrency fraud. To be regarded relevant, the title and abstract had to mention fraudulent behaviour linked to cryptocurrency technology. After completing the first round, the reviewers discussed disagreements and resolved them by consensus. Second, the two reviewers individually assessed the full texts of the articles to identify those that discussed cryptocurrency frauds and related empirical evidence. Any disagreements were resolved through discussion. This covered papers through June 2019. As this is a fast-moving field, the search was updated in November 2020. One reviewer (AT) conducted this subsequent search to capture articles released between June 2019 and November 2020, following the same process as the initial search.

In November 2020, one reviewer (AT) selected publications eligible for the grey literature scoping review in two steps. First, the reviewer screened the title and executive summary (or first section if none existed) of the publication for language, publication type, and relevance to cryptocurrency fraud. In addition, the reviewer searched the text of the source for ‘fraud’ and ‘scam’ and read the paragraph(s) including those words. To account for the fact that many sources did not have executive summaries, the reviewer adopted a permissive attitude at this stage; to be regarded as relevant, the content screened did not need to explicitly discuss fraudulent behaviour in detail. Rather, the reviewer included the publication if, from the content reviewed at this stage, the full text could reasonably be expected to discuss cryptocurrency fraud. Second, one reviewer assessed the full texts of publications meeting the initial criteria to identify those that discussed cryptocurrency frauds and related empirical evidence.

#### Data extraction process

Next, data were extracted from the included studies by one of the three reviewers. During the first round of the review, the data extraction form was tested by having the first two reviewers independently code 25 of the included studies. Disagreements were discussed and the form was updated accordingly. The final version of the data extraction form (see Table 2) was then used to extract information from all studies.

**Table 2:** Characteristics of the literature extracted

Item label	Description	Example
Author(s)	Author’s last name/ First author’s last name plus the abbreviation et al. as appropriate	Doe/ Doe, et al
Year	Year of publication (YYYY)	1999
Publication type	Type of publication ranging from theses to peer-reviewed papers	Monograph
Research area	Affiliated field of research of the publication	Computer science
Data type	Label of the type of empirical evidence	Account information
Data analysis method	Label of the data analysis method	Machine learning
Cryptocurrency technology	Name of the of cryptocurrencies related to scams	Ethereum
Fraud types	Label of the cryptocurrency fraud type(s)	Ponzi scheme
Definition: fraud types	Publication fully/partially/not reported definitions for the discussed fraud types	Fully reported

### Results

#### Study selection and characteristics

**Fig. 1:**
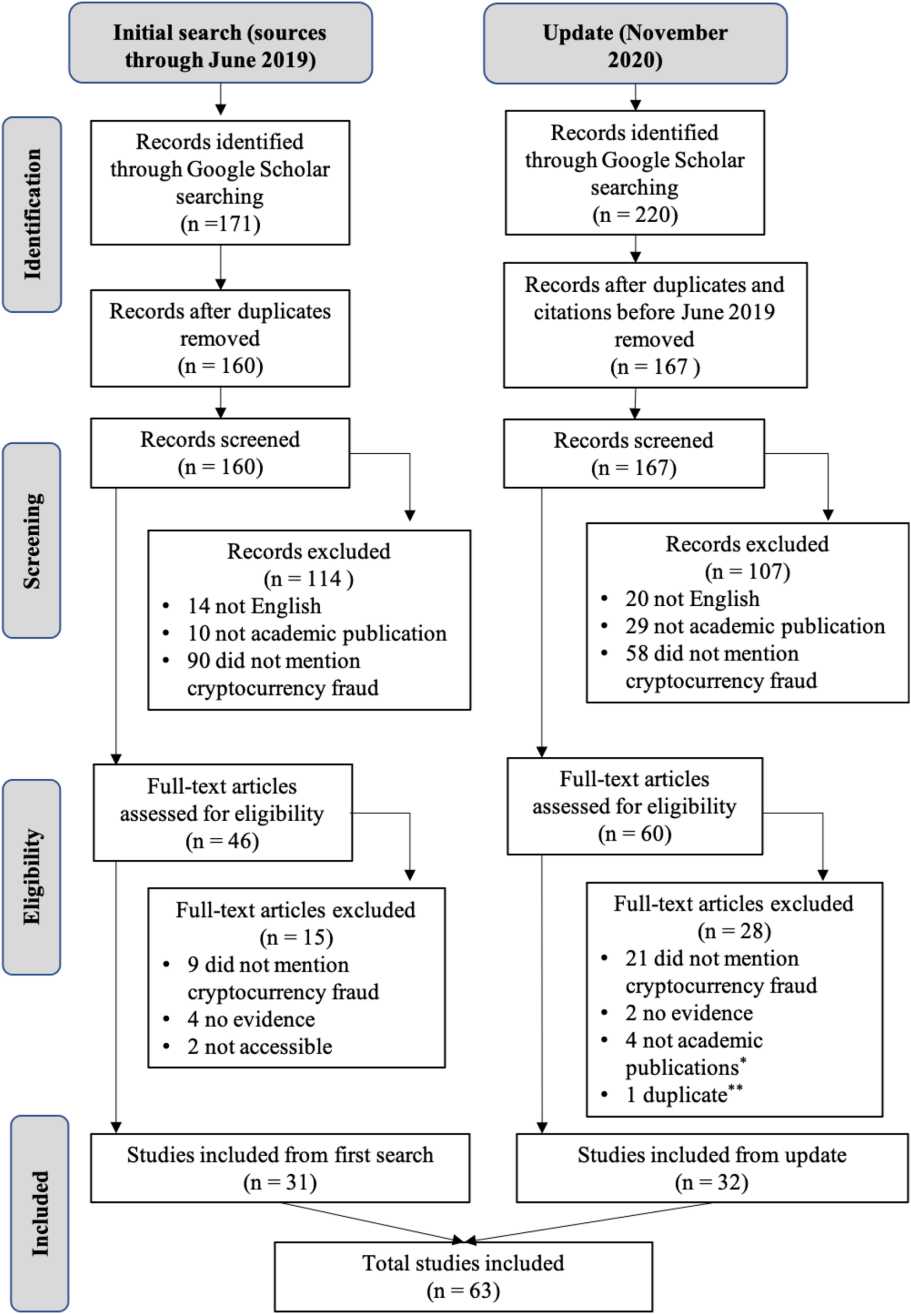
PRISMA-ScR flow diagram. *Articles were published on electronic pre-print service SSRN and appeared to be academic in nature from screening their titles and abstract but, upon full-text examination, were excluded. **One article was included in the initial search as a pre-print, but had since been formally published and was, therefore, excluded during the updated search as a duplicate

Figure 1 shows the PRISMA-ScR flow diagram (Moher et al., 2009), which summarises the study selection process for the two academic searches. GS identified 171 citations in the initial search and 220 in the November 2020 update. After removing duplicates, we screened 160 publications during the initial iteration and 167 in the update (i.e., a total of 327 unique publications). Based on the title and abstract, we excluded 114 records from the initial search and 107 from the second. As shown in Fig. 1, a small number of studies were excluded because they were published in a language other than English, or because they were not academic publications, but most of those that were excluded did not address the topic of cryptocurrency fraud; rather, they were focused on topics like the technological and regulatory challenges of cryptocurrency ecosystems.

This left 46 studies from the initial search, of which 15 were excluded following full-text assessment. In total, 31 studies met the inclusion criteria and are included in the review from the initial search. We evaluated the full text of 60 publications during the November 2020 update, of which 32 were ultimately included. The four articles ultimately deemed not to be academic in nature were published on the electronic pre-print service SSRN; they appeared to be academic publications from their titles and abstract but, upon full-text examination, were excluded. The duplicate article was included in the initial iteration of the search as a pre-print but had since been formally published. The content had not changed and, therefore, it was excluded as part of the second full-text review. Overall, 63 total studies met the inclusion criteria and are included in this scoping review (see Appendix 1: Table 3 or https://osf.io/7w9mu/?view_only=c9ad3a1e2ed54dae9b1a0fc2807f144f for summary details of these studies).

**Fig. 2:**
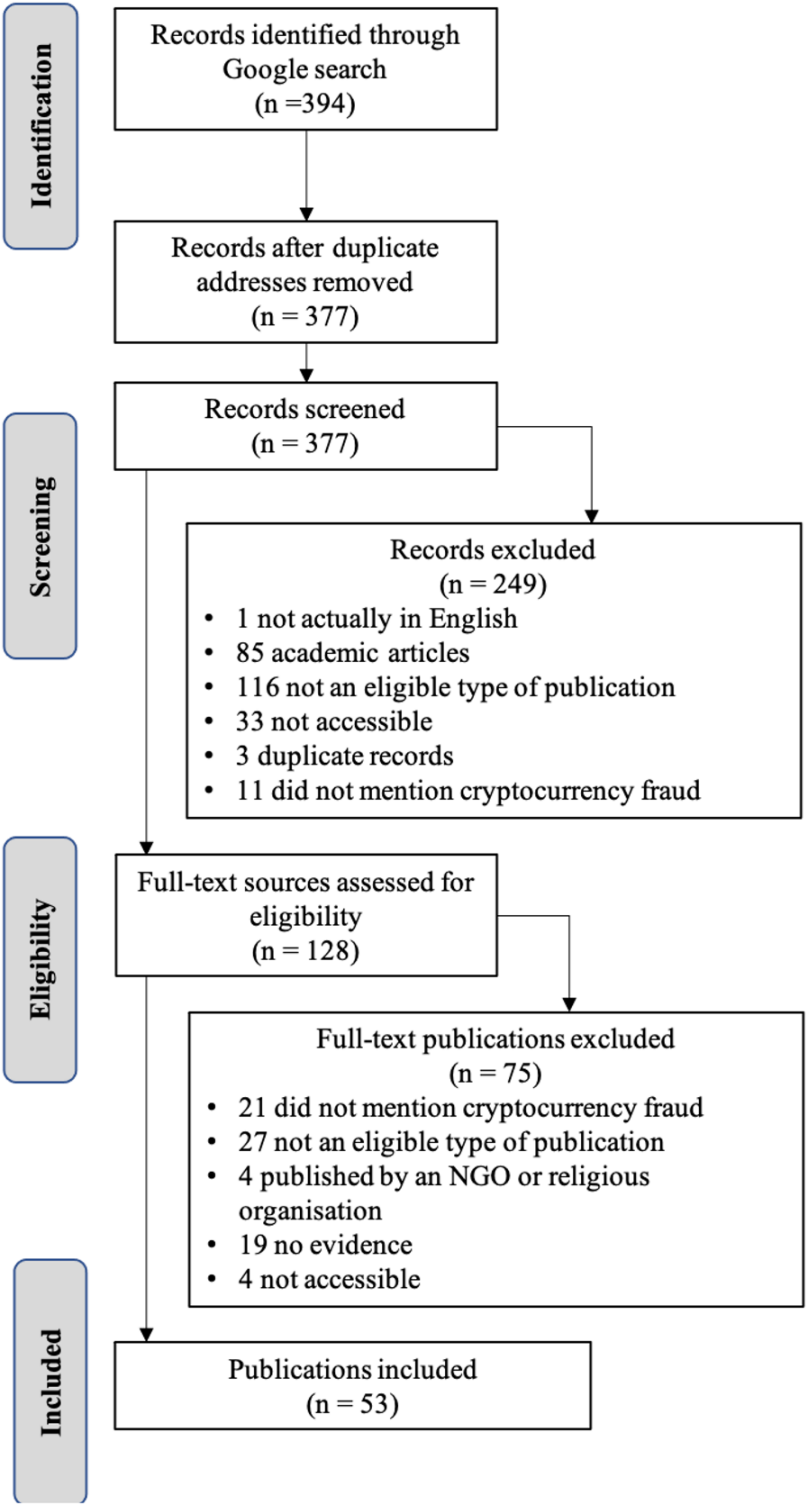
PRISMA-ScR flow diagram for grey literature selection (search conducted November 2020)

Figure 2 summarises the publication selection process for the grey literature. We identified 394 records through the Google search. After removing duplicate web addresses, we screened 377 publications. Based on the title and summary (or the first section of the documents), we excluded 249 records. Of these, one was published in a language other than English; 85 were academic publications; and 116 were ineligible types of publications.^4^ Thirty-three sources were either not accessible or were excluded because opening them posed a privacy or security risk. Eleven sources did not address the topic of cryptocurrency fraud and three were, upon further inspection, duplicates.

This left 128 sources, of which 75 were excluded following full-text assessment. Fifty-three studies met the inclusion criteria and are included in the review (see Appendix 2: Tables 4 and 5 or https://osf.io/7w9mu/?view_only=c9ad3a1e2ed54dae9b1a0fc2807f144f for summary details of these studies).

#### Types of fraud

In this section, we identify the specific forms of cryptocurrency fraud discussed and the definitions thereof.

**Fig. 3:**
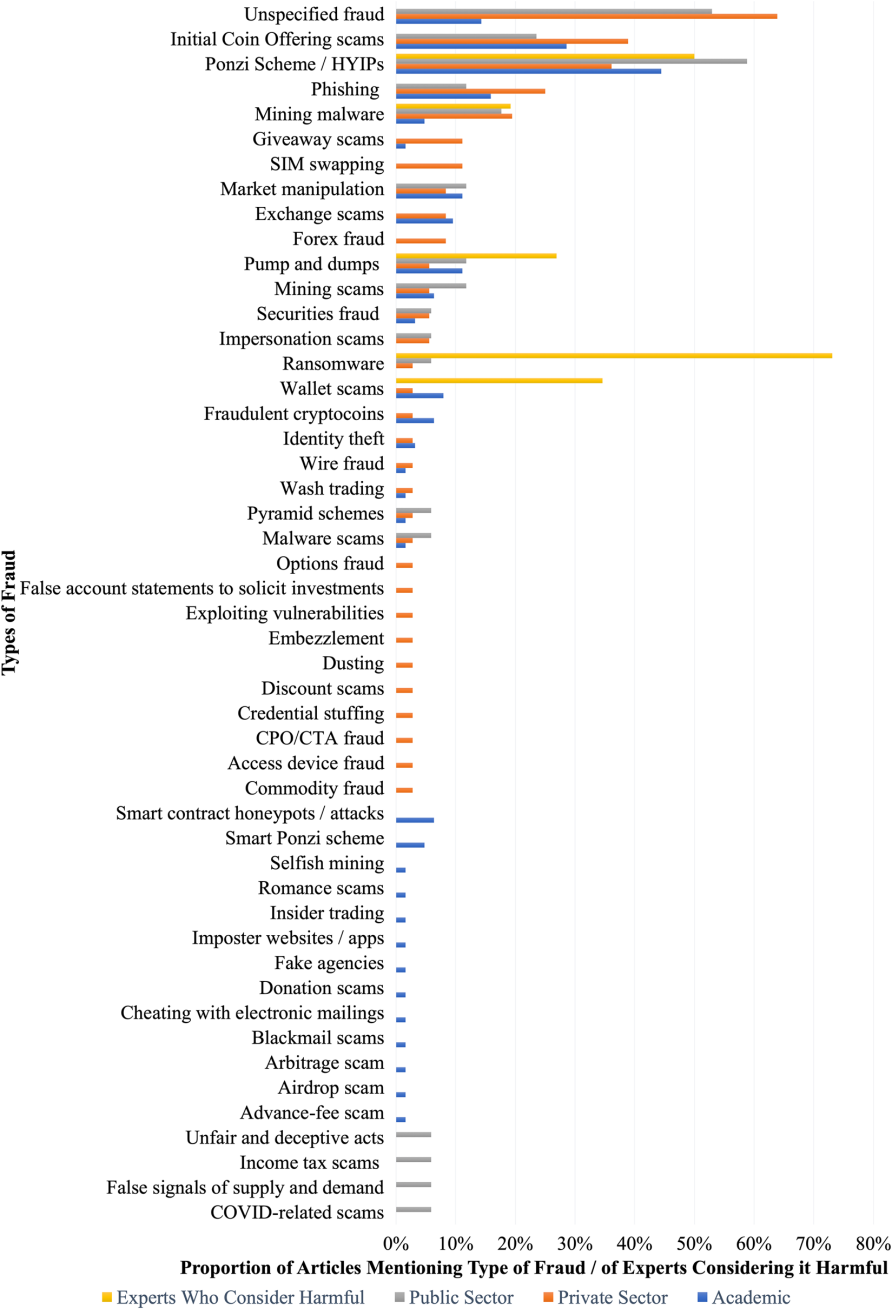
Number of publications and expert consensus exercise participant votes per fraud type. *CPO/CTA fraud is an abbreviation for Commodity Pool Operator or Commodity Trading Advisor fraud. For more details, see Appendix 3 or https://osf.io/7w9mu/?view_only=c9ad3a1e2ed54dae9b1a0fc2807f144f

The academic literature identified 29 different types of cryptocurrency fraud. Figure 3 lists all fraud types identified in the literature and the proportion of publications that discussed them, while Appendix 3: Table 6 provides descriptions of the offences.^5^ It is worth noting that in the literature reviewed, authors did not always clearly define or differentiate among types of fraud. Specifically, out of the 63 included academic studies, 30 (47.6%) fully reported definitions for all types of fraud discussed, while almost the same number of publications (33; 52.4%) did not. This lack of conceptual clarity is unfortunate as it impedes understanding of how the frauds are committed and how we might address them. For the benefit of the reader, where necessary, Appendix 3: Table 6 includes definitions derived from additional sources.

Academic publications most frequently referred to Ponzi schemes and (synonymous) high yield investment programmes (HYIPs). These scam types were discussed in 44.4% of the included studies. Eighteen (28.6%) publications analysed scams involving initial coin offerings (ICOs). Ten analyses (15.9%) covered phishing scams and nine (14.3%) discussed unspecified types of fraud. Seven (11.1%) studies covered pump-and-dump schemes and market manipulation. Six (9.5%) studies looked at exchange scams and five (7.9%) at scam wallet services. Four papers (4.8%) discussed each of the following types of fraud: fraudulent cryptocoins, smart contract honeypots / attacks, and mining scams. Three publications (4.8%) discussed mining malware and the same number addressed smart Ponzi schemes. Two (3.2%) publications discussed securities fraud and identity theft. Sixteen fraud categories were only mentioned in a single (1.6%) publication each. The second iteration of the search identified 17 new types of fraud from the literature.

Altogether, 36 of the grey literature publications came from private sector companies. These publications identified 32 different types of cryptocurrency fraud, 14 of which were not identified in the academic literature. Figure 3 shows these and the proportion of publications that discussed them, while Appendix 3: Table 7 provides descriptions of any offences which were not previously defined in the academic literature.^6^ Even more so than in the academic literature, authors did not clearly define or differentiate between types of fraud. Specifically, only four of the 36 studies (11.1%) fully reported definitions for all types of fraud discussed.

Most private sector studies (63.9%) referred to some unspecified type of fraud or scam. Fourteen (38.9%) publications analysed scams involving ICOs and 13 (36.1%) discussed Ponzi schemes or HYIPs. Nine (25.0%) studies covered phishing and seven (19.4%) covered mining malware. Four studies (11.1%) looked at SIM swapping, which did not appear in the academic literature, and which is defined in Appendix 3: Table 7. Four studies (11%) also discussed giveaway scams. Three studies (8.3%) discussed market manipulation, forex fraud, and/or exchange scams. Two studies (5.6%) looked at impersonation scams, mining scams, pump-and-dumps, and/or securities fraud. Eighteen fraud categories were mentioned in a single publication each (2.8%).

Seventeen different types of cryptocurrency fraud were identified in the public sector literature. Complete descriptions of these were provided for only four (23.5%). Definitions of frauds covered only in the public sector literature can be found in Appendix 3: Table 8.^7^

The most frequently discussed were Ponzi schemes and HYIPs, which were covered in 58.8% of studies. This was followed closely by coverage of undefined or general fraud and scams (nine publications, 52.9%). Four publications (23.5%) addressed ICO fraud and three (17.6%) covered mining malware. Two studies each (11.8%) covered mining malware, pump-and-dumps, phishing, mining scams, and/or manipulation and market abuse. Nine types of fraud were only mentioned in a single (5.9%) publication each.

## Expert consensus exercise

A recurring issue in the literature reviewed was the absence of clear definitions of the fraud types identified. In addition, few publications included any assessment of the level of risk presented by the offences: while the existence of a publication on a particular topic is evidence of research effort, this does not necessarily imply that the problem is severe or important. To address this gap, we held a 1.5-day ‘sandpit’ exercise in which we sought to elicit expert opinion on these issues from a diverse group of stakeholders in the field. The aim of the event was to complement our academically-focussed scoping study by obtaining subjective views on a range of issues: the cryptocurrency frauds identified in the literature, any additional threats not present in the literature, the potential for future crime, and the challenges and opportunities participants experienced or anticipated.

The sandpit activity was held in June 2019, with 27 high-profile representatives from the tech industry, the financial sector (HSBC, Nasdaq, Facebook), international financial intelligence units (from the UK, the Netherlands and Australia), law enforcement (Metropolitan Police, City of London Police, Her Majesty’s Prison and Probation Service, National Crime Agency, Defence Science and Technology Laboratory), as well as the World Bank and academic researchers (UCL, Georgia State University, Australian National University, Imperial College London). Findings from the first iteration of the scoping review were presented to inform the activity. However, to maximise the information provided by attendees, the findings from the scoping study were introduced as a way of providing an overview of the problem as it was represented in the published literature (at that time) and to frame the discussions, rather than a point of reference intended to limit their thinking.

Hereafter, we provide an overview of the planning considerations and structure of the event, as well as a summary of the activities,^8^ their results, and key conclusions.

### Methods

The event commenced with a general introduction and a presentation of the preliminary findings of the initial scoping review. All participants introduced themselves and described what they saw as the key problem with cryptocurrencies for their sector/area and what they thought were the key drivers and inhibitors of the adoption of cryptocurrencies.

Next, to ensure a common understanding of the overall topic, two invited talks were given: one providing an overview of the blockchain and cryptocurrencies, and the second offering an empirical example of a cryptocurrency fraud; in this case, pump-and-dump schemes (based on Kamps & Kleinberg, 2018).

Two group *problem planning* exercises formed the core part of day one. In predetermined groups (allocated to include members of each sector to facilitate cross-pollination of ideas), participants first engaged in the development of a fraud strategy. Their task was to devise a fraud/crime scheme with cryptocurrencies. Groups started in pairs to develop initial ideas, then joined another pair to decide on one fraud activity and further developed that idea in their group. These findings were then presented in a plenary setting. Next, in the second problem planning phase, each of the groups was assigned a fraud scheme from another group and had to devise mitigation steps. Specifically, the groups were tasked with thinking about what is already in place to mitigate cryptocurrency-related crime, what is needed for better mitigation efforts, and how they would address their allocated problem. As in the first problem planning phase, each group presented their mitigation ideas to the wider audience.

Day two was dedicated to analysing the problems identified. Participants were again allocated to groups, different from those of the first day to ensure that everyone interacted with as many others as possible. In roundtable discussions, the groups focused on the core problems identified on day one and were asked to indicate (on a scale from 1 = very low to 7 = very high) how harmful, profitable, feasible and defeatable they found each of the problems. These judgments were made using interactive polling software that allowed them to access the poll with their smartphone and see the (anonymised) results in real-time. Expert opinions concerning these four facets were particularly pertinent given the absence of such insights in the literature.

After a brief discussion of the findings, we proceeded in a plenary setting and focused on the wider problems associated with cryptocurrencies identified on day one. All participants were again asked to use the polling software to rank the issues according to their relevance. The problem analysis exercise closed with a ranking of the importance of the drivers and inhibitors identified during the introduction of the first day.

The activities of the event closed with three further questions about the future; another area about which the literature provided limited insight, and for which the answers to these questions were, therefore, critical to guiding future academic research. Specifically, we asked participants individually (using the polling software): (1) what they expected to see in the cryptocurrency space in ten years’ time; (2) what they definitely did not expect to happen; and (3) what would be needed to better address the potential criminal exploitation of cryptocurrencies in the broadest sense.

### Summary of findings

#### Problem planning exercise and analysis of issues

The initial *problem planning* exercise resulted in the identification/ production of various problem scenarios by the invited participants, as follows^9^:

- Fake crypto wallets;
- Pump-and-dump schemes;
- Investment scams (includes ICO scams, Ponzi schemes, and HYIPs);
- Cryptojacking (mining malware); and
- Ransomware.

#### Problem analysis and evaluation

The problems identified in the scenario planning group exercises were discussed, and participants were asked to rate them (on a seven-point scale such as: 1 = not harmful at all to 7 = very harmful) regarding their harmfulness, profitability, feasibility, and defeatability. To capture their confidence in ratings made, participants were also asked to indicate the certainty in their judgments (1 = low certainty to 7 = high certainty). Participants completed the task individually using polling software.^10^

To facilitate comparison with the results of our scoping review, the proportion of participants in this exercise who identified each type of fraud as a source of harm in the cryptocurrency space is also included in Fig. 3. Figure 3 displays the proportion of participants who rated the harmfulness of each of these as ‘5’, ‘6’, or ‘7’. The expert consensus exercise participants did not differentiate between ICO scams and other HYIPs, but we have displayed their responses under the latter, more general category. There are clear discrepancies between the extent to which certain threats were identified by experts and the frequency with which they appear in the literature. While Ponzi schemes and other scams were common in both, two of the primary threats identified by experts—ransomware and wallet scams—were among those which only received modest attention in the literature. In contrast, the level of published material concerning issues such as phishing appeared disproportionate to its perceived risk.

**Fig. 4:**
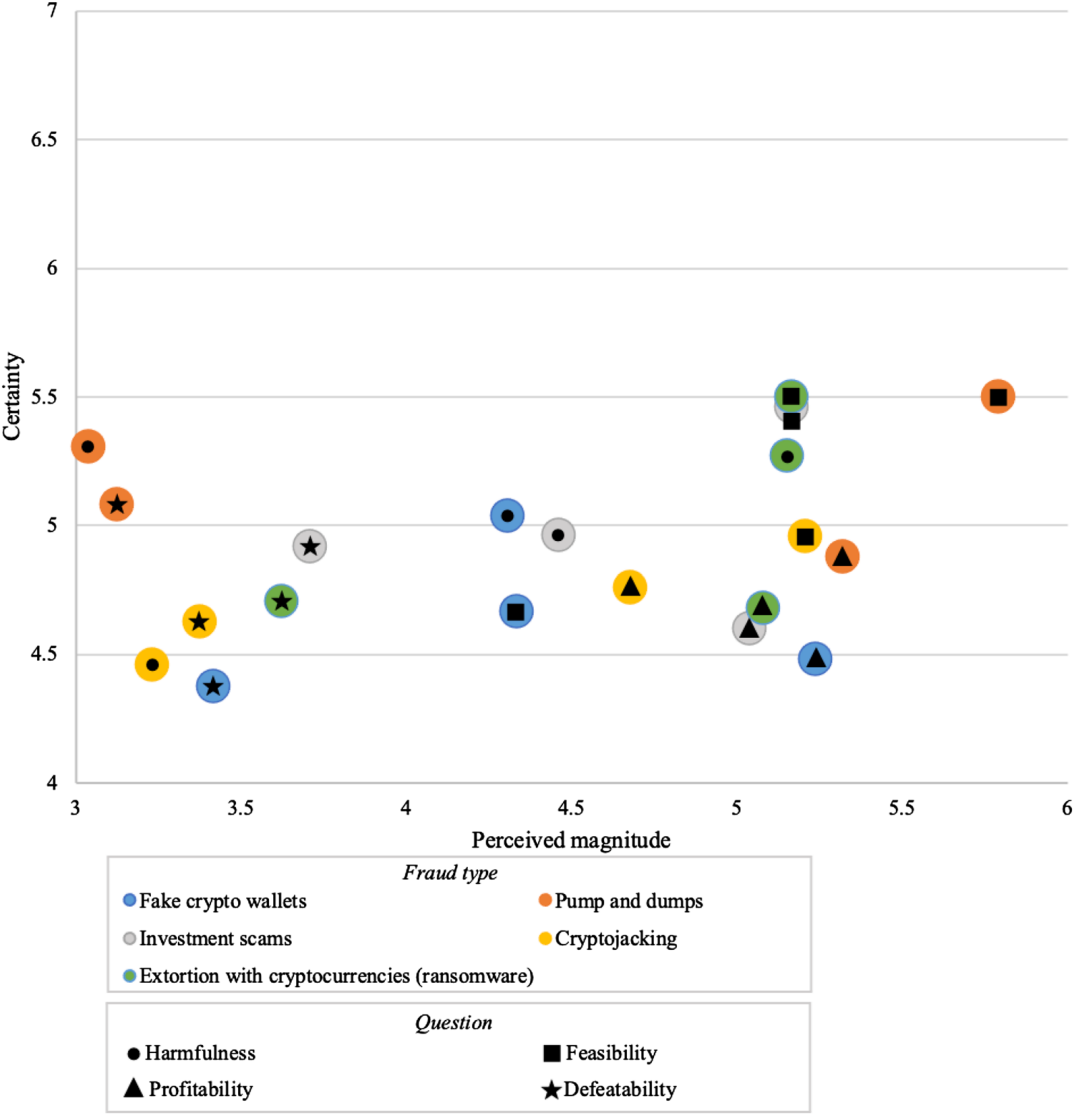
Problem analysis on the harmfulness, profitability, feasibility, and defeatability dimensions (horizontal axis; judgment certainty on the vertical axis)

The aggregate results for all four dimensions (averaged across participants) are shown in Fig. 4. For each of the dimensions, the graph can be interpreted in much the same way: for example, the offences that participants perceived to be most harmful and for which they were the most certain of their judgement are in the top right of the figure.

Overall, the problems discussed scored higher on their feasibility than they did on their defeatability. The tendency for participants to perceive defeating these problems as more difficult than devising the scams was a recurring topic during the exercise. While most offences were perceived to be profitable, participants were divided in terms of the degree of harm they posed. For all dimensions considered, participants expressed varying degrees of (un)certainty, suggesting a need for more knowledge on these offences.

In terms of the most highly ranked threats, pump-and-dump schemes and ransomware were perceived as the most profitable and most feasible. These were also the two offences for which participants tended to express the most confidence in their answers. Interestingly, perceptions differed for the perceived harm associated with pump-and-dump schemes, which were seen as the overall least harmful issue. An explanation for this disparity could be the perceived lack of victims for pump-and-dump operations. During the activity, participants voiced concerns that pump-and-dump operations are a known risk of which all cryptocurrency market participants should be aware.

#### Final three questions about the future

Participants emphasised the demand for better collaboration across sectors to address the problems discussed. Some highlighted the need for better sharing of intelligence and collaboration between cryptocurrency exchanges and public institutions. Unfortunately, others highlighted the same things as being unlikely to happen. Such diversity of opinion was also present for broader scenarios: specifically, while some anticipated a fully cashless society and easy-to-use cryptocurrency payments for everyday items in the next ten years, others saw the same scenario as unlikely.

Collectively, the findings demonstrate that we are at an early stage in thinking about future problems and scenarios involving cryptocurrencies. An area of agreement was the need for better collaboration between sectors since neither the private sector, nor the law enforcement or financial intelligence units can address the problem alone.

## Discussion

This scoping review represents the first summary of available research on cryptocurrency fraud and definitions of the types of fraud identified by the research. Findings suggest research on cryptocurrency fraud is rapidly developing both in volume and breadth. Criminals appear to be rapidly expanding into other areas of fraud and research has, so far, been unable to keep up. While it is unwise to conflate the volume of research on particular types of fraud with the magnitude of offending, the existence of empirical evidence of a number of different types of fraud about which there is little academic research supports this assertion. Key findings and limitations are discussed below, emphasising the need for further research on newly identified areas of cryptocurrency fraud and collaboration across stakeholders.

### Cryptocurrency fraud as a cyber-enabled crime

Most sources portrayed cryptocurrency frauds as cyber-enabled frauds. Cyber-enabled crimes involve perpetrators using information and communication technologies to magnify the scale and reach of offences that could also be committed offline (McGuire & Dowling, 2013). In describing cryptocurrency frauds, researchers often refer to traditional financial frauds like Ponzi schemes (Bartoletti et al., 2018; Reddy & Minaar, 2018; Securities & Exchange Commission, 2013), market manipulation, and pump-and-dump schemes (Anderson et al., 2019; Chen et al., 2019a, 2019b, 2019c, 2019d). These types of fraud are not new—Charles Ponzi first committed his namesake fraud in the 1920s, promising high returns for investments in stamps (Frankel, 2012). Pump-and-dump schemes have similarly plagued the stock market for centuries (Kamps & Kleinberg, 2018).

While the underlying characteristics of these frauds remain unchanged, implementation mechanisms have evolved. For example, there are significant parallels between ICOs and initial public offerings (Barnes, 2018; Baum, 2018) but, rather than shares being offered via a stock exchange, ICOs raise funds through the blockchain. Furthermore, smart contracts have transformed the way Ponzi schemes can be executed (Bartoletti et al., 2017; Chen et al., 2018a, 2018b, 2019a, 2019b, 2019c, 2019d). Overall, however, the literature points to significant similarities between cryptocurrency frauds and traditional financial frauds (or at least the academic imagination conceptualises it this way).

Research refers to cryptocurrency frauds as cyber-dependent comparatively less often. Cyber-dependent crimes are offences that are only able to be committed using information and communication technologies (McGuire & Dowling, 2013). Crypto-mining and wallet and exchange service frauds can be categorised as such. For example, crypto-mining frauds involve malware which uses a victim’s computer to mine cryptocurrencies for the offender (Anderson et al., 2019; Conley et al., 2015). In the case of wallet and exchange service frauds, fraudsters impersonate legitimate versions of such services, only to later steal money from victims (Pryzmont, 2016; Samsudeen et al., 2019; Vasek, 2017; Vasek & Moore, 2015). These are, perhaps, the only two types of fraud identified which could be considered strictly crypto-dependent, as opposed to other crimes, such as ransomware, which—while cyber-dependent—are merely facilitated by cryptocurrencies. While these frauds were not particularly prominent in the literature, they illustrate how new technologies facilitate novel crime opportunities, not just in terms of the technologies themselves, but also through the supplementary services created alongside them. As cryptocurrency use becomes more mainstream, new cyber-dependent methods of fraud may emerge. There is particular potential for this to occur as the decentralised finance industry further develops (Schär, 2021).

The fraud types identified in the expert consensus exercise were more evenly split in terms of cyber-enabled and cyber-dependent crimes. Three of the crimes discussed (fake cryptocurrency wallets, cryptojacking, and ransomware) are cyber-dependent, while pump-and-dump schemes and investment scams are cyber-enabled.

### Definitions in the literature

Insufficient reporting of definitions in the literature across all sectors (but especially in non-academic literature) was observed. One of the primary contributions of this study is, therefore, to provide a comprehensive list of definitions of the types of fraud identified in the literature (see Appendix 3 or https://osf.io/7w9mu/?view_only=c9ad3a1e2ed54dae9b1a0fc2807f144f). In some cases, for example in legal sector sources (both academic and non-academic), this may be due to disciplinary norms. Legal scholars tend to assume fraud definitions refer to their statutory definition, which would be known to their intended audience.

There were also different definitions of certain types of fraud across the literature. For example, ‘credential stuffing’ was defined slightly differently in the private sector literature than its categorisation in academic literature (Krone et al., 2018; Navarro, 2019). Similarly, one academic study defined all ‘malware’ as ransomware (Xia et al., 2020a). Furthermore, across the grey literature, types of crime ordinarily not considered fraud, per se—such as ransomware, embezzlement, and other malware—were all categorised as such.

In some cases, it was more difficult to synthesise the types of fraud due to disparities in definitions. For example, one article considered ‘imposter websites and apps’ an issue; it was unclear if the author intended this to be categorised as distinct from phishing or if this was another way to describe the same criminal act (Scheau et al., 2020). Similarly, one paper referred to ‘unfair and deceptive acts’ as a type of fraud but failed to define it. Without a definition and an understanding that this is likely to refer to the Federal Trade Commission Act (Federal Trade Commission Act, 2012), this could easily be misinterpreted as ‘unspecified fraud’ (Scott, 2020).

There were different levels of depth in definitions across sectors. For example, public sector literature included ‘market abuse’ when discussing ‘market manipulation’ (HM Treasury et al., 2018). Another public sector article referred to COVID-related scams very generally, while an academic article split them into several different categories (NHS National Services Scotland, 2020; Xia et al., 2020a). Finally, some publications referred to individual types of fraud that were actually sub-categories of other types of fraud. For example, pump-and-dump schemes are one type of market manipulation.

### Development of academic research over time

The most discussed types of fraud (Ponzi schemes/HYIPs and ICO scams) remained the same between the first iteration of our academic literature review and the update. The third most discussed was phishing, which was newly identified in the research during the second iteration.

Overall, 17 new types of fraud were identified in the updated literature review, all of which were cyber-enabled crimes. Since they were all cyber-enabled crimes (and not ‘new’, cyber-dependent crimes), it was surprising that these went unidentified in earlier literature. It is unclear if criminals are adapting to enforcement efforts and committing new types of fraud or if the research is simply ‘catching up’. Interestingly, besides phishing, only two other newly identified types of crime—securities fraud and identity theft—were mentioned in more than one publication.

Some of this change could be due to the fields responsible for publishing these papers. In the first iteration, there were more computer science papers; they would be less likely to pick up on legal issues like securities fraud.

There is a clear need for more research on these ‘newer’ types of fraud. This need is further supported by the expert consensus exercise participants’ varying degrees of (un)certainty about harmfulness, profitability, feasibility, and defeatability of the offences discussed. The volume of research is growing rapidly—this review identified more eligible publications published in the last year than in the first several years included in the first iteration of the academic literature review. Considering this rapid development, a follow-up expert consensus exercise could be useful, which we discuss in more detail below.

### Differences among sectors

One of the primary conclusions from the sandpit exercise was the need for further collaboration among stakeholders. To address this, the updated version of this scoping review expanded to include grey literature sources.

Private sector literature was less specific in its discussion of fraud than other sectors—‘unspecified fraud’ was discussed most often. The prevalence of the ‘unspecified fraud’ categorisation also highlights the lack of transparency in many private sector publications, in both their methods and conclusions. ‘Unspecified fraud’ was followed by the same three most ‘popular’ types of fraud as in the academic literature—ICO scams, Ponzi Schemes / HYIPs, and phishing.

New types of fraud (e.g., SIM swapping, forex fraud, and securities fraud) were more commonly discussed in the grey literature than in the academic literature. This was true even though the academic review was recently updated. Notably, SIM swapping, forex fraud, and impersonation scams were completely absent from the academic literature. The remaining ‘new’ crimes identified in the private sector literature were only mentioned in one publication each and can be categorised as cyber-enabled crimes. These may, indeed, be crimes that have only recently emerged in the cryptocurrency space. In the public sector literature, Ponzi schemes, unspecified fraud, and ICO scams were the most frequently discussed types of fraud. Phishing was also frequently discussed in the public sector literature.

### Focus of research and expert consensus exercise and prioritising future research

Across all sectors of published research, Ponzi Schemes/HYIPs and ICO scams were discussed the most often but were not considered particularly profitable or feasible by sandpit participants. It is possible that more research into—and a greater understanding of—these scams has made them less feasible, or at least has led to them being perceived as such. There was less certainty among participants surrounding the profitability of investment scams; future academic research (and collaboration with other stakeholders) could serve to reduce this uncertainty.

There could also, however, be a mismatch between research and practice. For example, our experts perceived crimes like ransomware and fake crypto wallets as profitable. Prior academic research has shown that ransomware, in particular, was not particularly so (Conti et al., 2018; Vasek et al., 2017). However, the applicability of this academic work might be limited by its age, as more recent, private sector sources have suggested ransomware has been increasing in recent years and that it has the potential to be very profitable and harmful (Chainalysis, 2021; CipherTrace, 2020). Beyond highlighting the need for further academic research on the impact of ransomware, this discrepancy emphasises the need for collaboration among stakeholders and academia in developing research agendas.

As noted by the expert participants of our sandpit activity, further collaboration is necessary across sectors to prioritise future research into cryptocurrency fraud. To facilitate this, a follow-up sandpit-style activity, informed by the updated and expanded scoping review, is recommended. It would be useful to gain experts’ insight into the harmfulness, feasibility, profitability, and defeatability of some of the ‘new’ cyber-enabled crimes we identified in the literature (e.g., securities fraud, identity theft, and wire fraud). Since the number of types of frauds has significantly increased, and many types of fraud were only mentioned in a single study, this is crucial to prioritising future research. The follow-up exercise should include broad participation from a variety of sectors to get a more comprehensive view of the current and future cryptocurrency-based fraud landscape. Finally, an emphasis on consensus surrounding definitions of various types of fraud would be useful. This could ultimately lead to the collaborative development of standards in the field, which could help prevent future frauds.^11^

### Limitations and Outlook

The limitations of this (and any) scoping review concern choices regarding the eligibility criteria and search strategy used. First, this scoping review was limited to GS. While it may be argued that using a single database may result in some publications being missed, we believe GS provides comprehensive coverage of the issues on which this review focuses. In designing the review, we conducted test runs across various databases, including ProQuest, Web of Science, and Scopus, with a combination of more general and more specific search terms. These searches returned a large volume of publications; however, many of the articles were merely news reports and the searches included many duplicates. In contrast to the other databases, GS returned the highest proportion of relevant, scientific work. While other similar studies (for example, Badawi and Jourdan (2020)) identified a higher volume of publications, this is primarily due to their broader inclusion criteria.

We tested multiple alternative search strings but found that these resulted in large volumes of irrelevant material being identified. The final search strings were chosen through trial and error, and were deemed to best reduce irrelevant material, while remaining processable in a reasonable timeframe. We ultimately restricted the GS search to exact phrases (as opposed to texts including the keywords in an unconnected manner) because test queries identified too many potential (but irrelevant) records when the search terms were less specific. We acknowledge that we may have excluded relevant studies that alternative search strategies would have detected. However, the fact that we uncovered such a large range and number of scams and frauds means the implications of this on our overall conclusions are likely to be minimal. Furthermore, GS lacks wildcard character functionality. However, in our experience, using wildcards on other databases primarily resulted in more noise, rather than better findings. Moreover, using wildcards reduces control over the search to a certain extent. We ultimately sacrificed some potential coverage for greater precision, control, reliability, and transparency.

We limited our scoping review to research published in English. Of the 160 records screened in the first iteration of our review, only 14 were excluded because they were not in English (i.e., 10.9% of the total records excluded after removing duplicates). In the second iteration, of the 167 records reviewed, 20 were excluded because they were not in English (i.e., 14.8% of the total records excluded after removing duplicates). While future studies may benefit from including non-English sources, we do not feel their exclusion from this study meaningfully affected our conclusions.

Besides ‘classical’ journal publications, we also categorised electronic pre-prints, industry reports, and theses as eligible publication types. Some may argue that such publications lack peer-review and are therefore of lower academic value. However, while they would not be subject to the official peer-review process, they are likely to have undergone informal academic review. Furthermore, regardless of their peer-review status, they serve as an indicator of research effort within the field, which is what we sought to measure. On the other hand, we excluded blogs and other sources which might more expeditiously capture what is currently happening or likely to happen in the future in terms of cryptocurrency fraud. We acknowledge that there is a trade-off between timely identification of types of fraud through these types of sources and credibility and verifiability. Many blog posts do not involve rigorous analyses or empirical evidence, may exaggerate claims for marketing purposes or shock value, and do not undergo any outside review (formal or informal). We sought to understand scams and frauds that have been verified (including via some level of peer review in the case of academic publications) and well-researched, rather than identify speculated, future-oriented insights. If we had primarily consulted blogs, many of the insights reported in this paper would not have been identified due to the lack of detail compared to formal articles and reports. We ultimately strove for a balance between prompt identification of frauds and credibility by including electronic pre-prints, theses, and the like.

We also excluded non-governmental organisations’ research from our grey literature review. However, only four of the 128 full texts screened were excluded for this reason. Finally, only one researcher updated the academic literature scoping review and conducted the grey literature review. Ultimately, we designed a rigorous process that was easily replicable and, therefore, do not consider it to have impacted our results.

As literature on this topic develops, further analysis of the literature’s insights—specifically on the harmfulness, feasibility, profitability, and defeatability of the frauds as well as information on whether they are increasing, decreasing or otherwise—would be pertinent. Unfortunately, it was not possible to glean such information from the literature in either iteration of this scoping review as it was largely absent. The absence of these insights in the literature was one motivation for including these factors in the expert consensus exercise but it would, ultimately, be useful have these perspectives from the literature as well. We invite further studies to analyse these frauds in more depth. Since developments in this field are fast-paced, we also recommend regular updates to this scoping review to maintain an accurate view thereof.

## Conclusions

In recent years, governments have reported an increase in frequency and scale of frauds involving cryptocurrencies. This review offers the first systematic study of research on what kinds of cryptocurrency fraud currently exist or are expected to exist in the future and, uniquely, systematically identifies expert practitioners’ assessments of these issues as well. The findings suggest scholarship on future problems and scenarios involving cryptocurrency fraud remains in its early stages, though research is rapidly developing (both in volume and scope). Even though many of the frauds identified in this research can be considered cyber-enabled (rather than cyber-dependent), the new ways in which they are being committed using cryptocurrencies necessitates future research.

Another notable finding was the lack of consistency (or existence at all) of definitions of the various types of fraud identified in the literature. One contribution of this study is, therefore, to provide definitions of all the types of fraud currently identified in the academic and grey literature (see Appendix 3 or https://osf.io/7w9mu/?view_only=c9ad3a1e2ed54dae9b1a0fc2807f144f). Further consensus surrounding these definitions could lead to the collaborative development of standards in the cryptocurrency sector, which would facilitate prevention of future frauds.

This work can help guide research agendas and activities aimed at translating research into practice. Ultimately, the study emphasises the need for better collaboration across sectors in prioritising future research on and mitigations of frauds involving cryptocurrencies to better address the problems identified.

## Data Availability

The datasets generated and analysed in the current study are available in the Open Science Framework repository, https://osf.io/7w9mu/?view_only=c9ad3a1e2ed54dae9b1a0fc2807f144f.

## Appendix 1

See Table 3.

**Table 3:** Academic papers included in this scoping review

Author	Date	Publication Type	Scientific Discipline	Data	Data Analysis Method		Cryptocurrency Technology	Fraud Types	Fraud Definitions
Anderson et al.	(2019)	Report	Computer science	Court cases	Descriptive statistics		Bitcoin, Bitconnect, OneCoin, Monero Tether	Fraudulent cryptocoins, Initial Coin Offering (ICO) scams, market manipulation, mining malware, Ponzi schemes	Partially reported
Badawi & Jourdan	(2020)	Journal Article	Engineering	Other publications	Systematic literature review		Bitcoin, Ethereum, Monero	General fraud, high yield investment programmes, pump-and-dumps, mining malware, phishing	Partially reported
Badawi et al.	(2020)	Conference Paper	Engineering	Scam addresses	Transaction analysis		Bitcoin	Mining scam	Fully reported
Barnes	(2018)	Conference paper	Accounting & finance	Anecdotes	N/A		Bitcoin, Ethereum, Litecoin, Tether	ICO frauds, market manipulation, pump-and-dump schemes	Partially reported
Bartoletti et al.	(2018)	Conference paper	Computer science	Transaction data	Machine learning algorithm		Bitcoin	Ponzi scheme	Fully reported
Bartoletti et al.	(2017)	Electronic pre-prints	Computer science	Transaction data	Quantitative & qualitative analysis		Ethereum	Smart Ponzi schemes	Fully reported
Baum	(2018)	Thesis	Accounting & Finance	Anecdotes	N/A		Billion coin, Bitcoin, Bitconnect, Bitfinex, Diamond Reserve Club, Ethereum, Giza, LoopX, Opair, PlexCoin, PonziCoin, REcoin, Tether	Exit scheme during or after ICO, fake ICO scheme, Ponzi schemes, pump-and-dump schemes	Fully reported
Boshmaf et al.	(2019)	Conference Paper	Computer science	Transaction data	BlockTag which uses vertical crawlers and clustering		Bitcoin	Ponzi scheme	Partially reported
Boshmaf et al.	(2020)	Conference Paper	Computer Science	User and service data	Linking accounts, and calculating monetary flows		Bitcoin but can be used on Litecoin, Namecoin, and Zcash and Others; MMM Ponzi scheme	Ponzi scheme	Partially reported
Chen et al.	(2020b)	Conference Paper	Computer science	Smart contracts	Machine learning model, LightGBM		Ethereum	Smart contracts honeypots	Fully reported
Chen et al.	(2020a)	Conference Paper	Computer science	Transaction records	Graph-based cascade feature extraction and lightGBM, a 'Dual-sampling Ensemble algorithm’		Ethereum	Phishing	Fully reported
Chen et al.	(2019a)	Conference paper	Accounting & finance; computer science	Transaction data	Singular value decomposition		Bitcoin	Market manipulation	Not reported
Chen et al.	(2019b)	Conference paper	Computer science	Transaction data	Improved Apriori Algorithm		Bitcoin	Pump-and-dump scheme	Fully reported
Chen et al.	(2018a)	Conference paper	Economics	Accounts and operation codes	Classification model		Ethereum	Smart Ponzi scheme	Fully reported
Chen et al.	(2019d)	Journal article	Computer science	Transaction data and operation codes	Classification model		Ethereum	Smart Ponzi scheme	Fully reported
Conley et al.	(2015)	Report	Law & legal studies	Anecdotes	N/A		Bitcoin	Mining malware	Not reported
Crowley	(2018)	Thesis	Law & legal studies	Anecdotes	N/A		Bitcoin	Ponzi scheme	Not reported
Dubois	(2019)	Thesis	Forensic & applied sciences	Survey	Statistical analysis and thematic, qualitative analysis		Bitcoin, Ethereum, Monero, Bitcoin Cash, Litecoin, Ripple, Dash, EOS, Dogecoin, Zcash, Cardano, Stellar, NEO, Scotcoin, Bitcoin Gold, Populous	General fraud	Fully reported
Duff et al.	(2018)	Report	Law & legal studies	Anecdotes	N/A		AriseCoin, ATM Coin, Bitcoin, Bitshares, BitUSD, CTR Tokens, Dogecoin, Litecoin, My Big Coin, REcoin	ICO scams	Not reported
Eastham	(2020)	Journal Article	Law	Anecdotal evidence	N/A		Tezos ICO	ICO scam	Not reported
Gandal et al.	(2018)	Journal article	Economics	Transaction data	Regression analysis		Bitcoin	Price manipulation	Not reported
Gao et al.	(2020)	Electronic pre-print	Computer science	Advertisements	Descriptive, lexical, popularity, and graphical analysis of creators and token holders; computational detection of airdrop scams		Ethereum	Counterfeit currency, airdrop scam, arbitrage scam	Fully reported
Hays	(2018)	Report	Law & legal studies	Anecdotes	N/A		Bitcoin	Ponzi scheme, market manipulation	Not reported
Henning	(2019)	Thesis / Electronic Pre-Print	Law	Court cases	N/A		Bitcoin, Libra, Dash, Ripple, Bitcoin Cash, NEO, BLV (Blockvest) Token, Argyle Coin, OneCoin, My Big Coins, PayCoin, Ethereum, Kin tokens, Grams	Wire fraud, securities fraud, Ponzi scheme, ICO scam, general fraud	Partially reported
Jiaying	(2020)	Electronic pre-print	Law	Case study	N/A		Ethereum, Qtum	ICO scams, pyramid schemes	Fully reported
Jung et al.	(2019)	Conference Paper	Computer Science, mathematics	Addresses	Weka, various classification algorithms—J48 decision tree, random forest, stochastic gradient descent		Ethereum	Ponzi schemes	Not reported
Kethineni et al.	(2019)	Journal article	Law & legal studies	News reports & magazines	Content analysis		Bitcoin, Monero	Ponzi scheme	Not reported
Lašas et al.	(2020)	Conference Paper	Mathematics	Information from wallets and transactions	K-means clustering, SVM, random forest classifier		Ethereum	General fraud	Not reported
Liu et al.	(2020)	Electronic pre-print	Media and communications, computer science, engineering	Other literature, anecdotal evidence	N/A		Bitcoin, Ethereum, Litecoin, Dogecoin, Zerocash, Monero, Zerocoin, Zcash, Dash, Cryptonote, Bytecoin, DigitalNote, Namecoin, Huntercoin, Myriadcoin, Unobtanium	Ponzi scheme, market manipulation, phishing, unspecified fraud	Partially reported
Murko & Vrhovec	(2019)	Conference Paper	Criminal justice and security	Online surveys	Statistical analyses		Bitcoin	Exchange scams, wallet scams, Ponzi scheme, mining scams	Fully reported
Murphy et al.	(2015)	Report	Law & legal studies	Anecdotes	N/A		Bitcoin	Ponzi scheme	Not reported
Najmy	(2019)	Journal Article	Law	Anecdotal evidence	N/A		Bitcoin	ICO scam	Fully reported
Navarro	(2019)	Thesis	Financial crime and compliance	Anecdotal evidence	N/A		Blockvest, BtCrush, Bitcoin, Etherum, Ringgold	Identity theft, phishing, romance scams, ICO scams	Fully reported
Ngai	(2014)	Thesis	Law & legal studies	Anecdotes	N/A		Bitcoin	Pump-and-dump scheme	Not reported
Nilsen	(2019)	Thesis	Computer science	Exchange data	Anomaly detection algorithm (Limelight)		GXS, Ethereum, Bitcoin, and various others (280 total anomalies detected for coins paired with BTC, some not specifically mentioned in paper)	Pump-and-dumps	Fully reported
Phan et al.	(2019)	Journal Article	Computer science	Tweets	Descriptive analysis, sentiment analysis, influence analysis		Bitcoin, Pincoin, Arisebank, Savedroid	Ponzi scheme, ICO scams, smart contract attacks, selfish mining	Fully reported
Phillips and Wilder	(2020)	Electronic pre-print (Conference Paper)	Computer science	Scam websites	DBSCAN clustering		Bitcoin and Ethereum	Advance fee fraud, phishing	Fully reported
Podgor	(2019)	Electronic pre-print	Law	Court case	N/A		Bitcoin, Etherum, ReCoin, Diamond	Securities fraud, unspecified fraud	Not reported
Pryzmont	(2016)	Thesis	Business	Anecdotes	N/A		Bitcoin	Fraudulent exchanges (service scam), Ponzi scheme	Fully reported
Reddy, et al.	(2018)	Journal article	Criminology	Anecdotes	N/A		Bitcoin	Ponzi scheme	Fully reported
Rognone et al.	(2020)	Journal Article	Business	Trading data, RavenPack News Analytics	VAR-X model		Bitcoin	General fraud	Not reported
Samsudeen, et al.	(2019)	Journal article	Computer science	Transaction data	N/A		Bitcoin	Fake agencies, scams involving exchanges	Fully reported
Șcheau et al.	(2020)	Journal article	Computer science; economics	Anecdotal evidence	N/A		Bitcoin, Pincoin, Plexcoin, IBCoin, Nelunx, Ruby-X, EOS, XRP, Ethereum, Litecoin, BitcoinCash, PlusToken	ICO Scams, unspecified fraud, imposter websites/apps, email scams, fake currencies, phishing, high yield investment programme, wash trading, fake wallets	Not reported
Semenihin, et al.	(2018)	Conference paper	Accounting & finance; law & legal studies	Anecdotes	N/A		Bitcoin	Cheating with electronic mailings, deception with cryptocurrency wallets, false ICO, non-real cryptocurrencies exchange stock	Not reported
Sureshbhai et al.	(2020)	Conference Paper	Computer science	Elliptic dataset	Sentiment analysis, long-short term memory classifier		Bitcoin	General fraud	Not reported
Torres, et al.	(2019)	Electronic pre-print	Computer science	Smart contracts	Systematic analysis		Ethereum	Honeypot	Fully reported
Toyoda et al.	(2019a)	Journal Article	Computer science	Bitcoin addresses	Supervised machine learning classifier		Bitcoin	High yield investment programme	Fully reported
Toyoda, et al.	(2018)	Conference paper	Computer science	Transaction data	Transaction history summarization		Bitcoin	High yield investment programme	Fully reported
Toyoda, et al.	(2019b)	Conference paper	Computer science	Transaction data	Time series analysis		Bitcoin	High yield investment programme	Fully reported
Toyoda, et al.	(2017)	Conference paper	Computer science	Transaction data	Transactions pattern analysis		Bitcoin	High yield investment programme	Fully reported
ur Rehman et al.	(2020)	Journal Article	Computer science	Other literature	Systematic literature review		Bitcoin, Ethereum, Ripple, Bitcoin Cash, EOS, Stellar, Litecoin, Tether, Bitcoin SV, Tron, Zcash, Monero, Dash	Market manipulation, insider trading, ICO scams, pump-and-dumps	Partially reported
Vasek	(2017)	Thesis	Computer science	Transaction data	Descriptive analysis		Bitcoin	Exchange scam, high yield investment programme, mining scam, scam wallet	Fully reported
Vasek, et al.	(2015)	Conference paper	Computer science	Transaction data	Descriptive analysis		Bitcoin	Exchange scam, high yield investment programme, mining scam, scam wallet	Fully reported
Vasek, et al.	(2018)	Conference paper	Computer science	Scammer and victim interactions	Survival analysis		Bitcoin	Ponzi scheme	Not reported
Vrazel	(2019)	Journal Article	Law	Anecdotal evidence	N/A		Denaro, Pluto Coin, LoopX, PlexCorps	ICO scams, phishing	Not reported
Waxenbaum	(2019)	Electronic pre-prints	Law & legal studies	Anecdotes	N/A		AriseCoin, Bitcoin, Bitshares, BitUSD, Dogecoin, Etherium, Litecoin	Fraudulent ICO	Not reported
Weber et al.	(2020)	Conference Paper	Computer science	Case studies	N/A		Red Pulse Token, Bee Token, Minerium Token	Social engineering (highly focused on phishing, but also includes pretexting, baiting), smart contract honeypots	Partially reported
Wu et al	(2020)	Journal Article	Computer Science	Phishing addresses	*trans2vec* network embedding algorithm*,* one-class SVM		Ethereum	Phishing, ICO scams	Fully reported
Xia et al.	(2020b)	Journal Article	Computer science	Scam domains and fake apps	Analysis of landing page source code and screenshots, VirusTotal labelling, Google Cloud Natural Language API, Vision API, descriptive analysis, clustering, monetary flows analysis		Not specified	Exchange scams	Partially reported
Xia et al.	(2020a)	Electronic pre-print	Computer science	Scams	Descriptive statistics and analysis, transaction analysis		CoronaCoin, COVID19 Coin,	ICO scams, giveaway scams, blackmail scams, malware scams, Ponzi scheme, donation scams	Partially reported
Xie	(2019)	Journal article	Law & legal studies	Anecdotes	N/A		Bitcoin	Ponzi scheme	Not reported
Yuan et al.	(2020)	Conference Paper	Computer science	Phishing addresses and transaction data	Network embedding method node2vec and SVM classifier		Ethereum	Phishing scams	Fully reported
Zetzsche, et al.	(2017)	Report	Law & legal studies	Anecdotes	N/A		Bitcoin	Fraudulent ICO, Ponzi scheme	Not reported

## Appendix 2

See Tables 4 and 5.

**Table 4:** Private sector sources included in this scoping review

Author	Date	Publication type	Scientific discipline	Data	Data analysis method	Cryptocurrency technology	Fraud Types	Fraud definitions
Akin Gump Strauss Hauer & Feld LLP	(2018)	Alert	Law	Court case	N/A	N/A	Unspecified fraud, Ponzi schemes, market manipulation	Not reported
Bolster	(2019)	Report	Artificial intelligence/cybersecurity	Anecdotal evidence	N/A	Libra, Cybertruck	Counterfeiting, fishing, fake ICOs, scam giveaways and discount scams	Partially reported
Burrus, Refinitiv	(2018)	Article	Financial services	Anecdotal evidence	N/A	Bitcoin	Fake ICOs, Ponzi schemes, pump-and-dumps	Partially reported
Chainalysis	(2020)	Report	Fintech	Global Crypto Adoption Index for all countries using analytics from SimilarWeb, data from LocalBitcoins and Paxful	'Global Crypto Adoption Index'	BCH, BTC, ETH, LTC, OMG, PAX, USDC, USD	Investment scams, other scams	Not reported
Chainalysis	(2019)	Report	Fintech	Ethereum addresses and users	N/A	Ethereum	Phishing schemes, Ponzi, ICO exit scams	Fully reported
Chernin et al., Cornerstone	(2020)	Report	Law	Court cases	N/A	Bitcon, Litecoin, My Big Coin, ATM Coin, CompCoin	Unspecified fraud, Ponzi schemes, CPO/CTA Fraud, options fraud, forex fraud, wash trading	Not reported
Ciphertrace	(2018)	Report	Fintech	Anecdotal evidence (other data was used for money laundering but for the actual fraud ones it was just anecdotal)	N/A	Ethereum	Spear phishing, SIM swapping, Ponzi schemes, fake ICOs	Partially reported
Cohen & Crance, Schnader Harrison Segal & Lewis LLP	(2018)	Article	Law	Court cases	N/A	Bitcoin	ICO fraud, unspecified fraud	Not reported
Coulter, Burr Forman LLP	(2018)	Alert	Law	Court case	N/A	N/A	Unspecified fraud	Not reported
Crowell & Moring	(2018)	Alert	Law	Court case	N/A	My Big Coin	Unspecified fraud	Not reported
Desai et al., Accenture	(2019)	Report	Consulting	Threats reported to Retail & Hospitality ISAC	N/A	Bitcoin, Ethereum	Scams, cryptocurrency mining malware, ICO scams	Not reported
Devonshires	(2018)	Report	Law	Court case	N/A	Bitcoin	Forex fraud	Not reported
Eversheds Sutherland	(2018)	Report	Law	Court case and anecdotal evidence	N/A	N/A	Securities fraud	Not reported
Financial Integrity Network	(2018)	Alert	Investigations and Compliance	Loss estimates from other source	N/A	Bitcoin, Monero, Dash, Counterparty, Ethereum, Openledger	ICO scams	Not reported
Global Blockchain Business Council / Accenture	(2020)	Report	Fintech	Court cases and statistics from other organisations	N/A	T-Chits, Bitcoin	Unspecified fraud, ICO fraud, phishing	Not reported
Goodlett, DLA Piper	(2020)	Article	Law	Court cases	N/A	N/A	High yield investment programme, unspecified fraud, mining scam	Not reported
Halverson, Mclean Roche Consulting	(2020)	Report (to Australian government)	Financial Services	Anecdotal evidence	N/A	Bitcoin, Ethereum, Ripple, Litecoin, Tehter, Bitcoin Cash, Libra, Monero, EOS, Bitcoin SV, Binance Coin,	Unspecified fraud, Ponzi scheme	Not reported
Hedrich et al., Marsh & McLennan Companies	(2018)	Report	Consulting	Anecdotal evidence	N/A	N/A	Unspecified fraud	Not reported
Insikt Group, Recorded Future	(2018)	Report	Security intelligence	IP addresses, social media profiles, case studies	N/A	Marine Chain, Interstellar / Stellar / HOLD / HUZU	Scams	Not reported
Krone, et al.	(2018)	Article	Law	Court cases and anecdotal evidence	N/A	Bitcoin, Monero	ICO scam, unspecified fraud, phishing, exchange scams, credential stuffing, pump-and-dumps, cryptojacking	Fully reported
LIFARS	(2018)	Article	Cybersecurity	Anecdotal evidence	N/A	Ethereum (Prodeum)	ICO scam	Not reported
Lim et al., Debevoise & Plimpton	(2019)	Report	Law	Court cases	N/A	N/A	Unspecified fraud, ICO fraud	Not reported
Lucking and Aravind, Allen & Overy LLP	(2019)	Chapter	Law	Court case and anecdotal evidence	N/A	Bitcoin	Thief impersonating federal employee, Ponzi scheme, unspecified fraud, pyramid scheme	Not reported
Mabille, FINATIC	(2020)	Report	FinTech	Anecdotal evidence	N/A	Bitcoin	Price suppression/ manipulation by whales, unspecified fraud, impersonation of celebrities, phishing	Not reported
Malyshev, et al., Reed Smith	(2018)	Article	Law	Court cases	N/A	Bitcoin	Unspecified fraud and manipulation, 'issuing false account statements in connection with soliciting investments in Bitcoin)	Not reported
McAvoy et al., Nixon Peabody	(2018)	Article	Law	Court case	N/A	Bitcoin	Ponzi scheme	Not reported
McGuire, Bromium	(2019)	Chapter	Cybersecurity	UK police data	N/A	Monero, Bitcoin	Cryptomining malware, ICO scams, Unspecified fraud	Fully reported
Musiala, et al., AICPA	(2020)	Report	Accounting	Court cases and anecdotal evidence	N/A	Bitcoin, Argyle Coin, PRO Currency, Libra,	Ponzi scheme, securities fraud, ICO scam, embezzlement, phishing, SIM swapping, dusting, mining malware, wire fraud, access device fraud, identity theft, ransomware	Partially reported
Parisi et al., Shearman & Sterling	(2018)	Article	Law	Court cases and anecdotal evidence	N/A	N/A	Commodity fraud	Not reported
PYMNTS.com / Tulioo	(2019)	Report	AML	Anecdotal evidence	N/A	Ripple, Bitcoin, Bitcoin Cash, Ethereum, Litecoin	Unspecified fraud, phishing and malware, exploiting vulnerabilities (hardware, software, etc.), high yield investment programmes, SIM swapping	Partially reported
Reik, Sprott Asset Management	(2019)	Article	Financial Services	Anecdotal evidence	N/A	Bitcoin	Unspecified fraud, ICO scams, fake exchanges,	Not reported
Rochemont, Institute and Faculty of Actuaries	(2020)	Report	Accounting	Anecdotal evidence	N/A	Bitcoin, OneCoin, MingoCoin, DasCoin / GreenPower	Forex scams, Unspecified fraud, Ponzi schemes, cryptojacking, SIM swapping	Not reported
Shearman & Sterling	(2018)	Article	Law	Court cases	N/A	My Big Coin	Unspecified fraud	Not reported
Štefanko, ESET	(2018)	Research whitepaper / report	Cybersecurity	Anecdotal evidence	N/A	Ethereum, Ada (on Cardano blockchain), Bitcoin, Dash, Litecoin, Dogecoin, Bitcoin Cash, Bitcoin Gold, Ripple, Monero	Fake cryptocurrency exchange apps/ phishing apps, fake cryptocurrency wallet apps, crypto-mining malware/ cryptojacking, fake crypto miners and free giveaways	Partially reported
Webroot	(2018)	Whitepaper	Cybersecurity	Anecdotal evidence	N/A	Monero, Bitcoin	Cryptojacking, giveaway scams on social media, other crypto scams	Partially reported
Wright, Duo Security	(2018)	Report	Cybersecurity	Twitter data (used previous study for labelling)	Machine learning algorithms: AdaBoost, Logistic Regression, Decision Trees, Random Forest, Naïve Bayes	N/A	Crypto giveaway scam	Fully reported

**Table 5:** Public sector sources included in this scoping review

Author	Date	Publication type	Scientific discipline	Data	Data analysis method	Cryptocurrency technology	Fraud types	Fraud definitions
Advertising Standards Authority and Committees of Advertising Practice	(2019)	Report	Advertising	Anecdotal	N/A	N/A	Unspecified fraud	Not reported
Australian Competition & Consumer Commission	(2018)	Report	Trade regulation	Scams reported to the ACCC	N/A	Bitcoin, Ether	Fake ICOs, pyramid schemes, ransomware, other scams	Fully reported
Australian Competition & Consumer Commission	(2020)	Report	Trade regulation	Scams reported to the ACCC and data from other Australian government agencies	N/A	Bitcoin, Ethereum, DSH, Ripple, Litecoin, EOS	Investment scams / Ponzi schemes, cloud mining cryptocurrency scams, celebrity endorsement scams	Fully reported
Capobianco, U.S. Department of Justice (to OECD)	(2019)	Memorandum	Legal	Court cases	N/A	Bitcoin	High yield investment programme, mining scam, cryptojacking,	Not reported
Elder & Rotunda, Texas State Securities Board	(2018)	Report	Financial Services	Investigations conducted	Descriptive analysis / N/A	Bitcoin, Ethereum, Litecoin, Ripple, Potcoin, Dogecoin, My Big Coin, Ponzicoin, Bitconnect Coins, DavorCoin, R2B	Unspecified fraud, high yield investment programme, fraudulent securities offerings	Not reported
Elwell et al., Congressional Research Service	(2014)	Report	Legislative	Court case	N/A	Bitcoin	Unspecified fraud, Ponzi scheme	Not reported
FSMA Belgium	(2020)	Alert	Financial Services	Consumer complaints	N/A	N/A	High yield investment programme, unspecified fraud	not reported
Garg et al., Department of Economic Affairs, Ministry of Finance, India	(2019)	Report and Bill	Finance	Anecdotal evidence, other studies	N/A	Bitcoin, Ethereum, Ripple, Cardano, Zerocoin (not mentioned in connection with fraud specifically)	Ponzi schemes, phishing, price manipulation	Not reported
HM Treasury, Financial Conduct Authority, Bank of England	(2018)	Report	Financial services	Qualitative consumer research, Action Fraud data	N/A	Bitcoin	Manipulation and market abuse, unspecified fraud, High yield investment programmes, fraudulent ICOs, cryptojacking, false signals of supply and demand (like wash trading, layering, spoofing), pump-and-dumps	Fully reported
Leuz (to U.S. Securities and Exchange Commission)	(2018)	Policy brief	Financial Services	Anecdotal	N/A	Proof of Weak Hands Coin	Pump-and-dumps, Ponzi scheme	Not reported
Lockhart & Nuesser, Innovative Research Group, Inc. (Report to Ontario Securities Commission)	(2018)	Report	Financial Services	Survey, anecdotal evidence	Descriptive statistics	Bitcoin, Ether, Litecoin, Bitcoin Cash, Ripple (in terms of ownership, not fraud)	ICO fraud	Not reported
Malta Financial Services Authority	(2019)	Alert	Financial Services	Anecdotal	N/A	N/A	Unspecified fraud	Not reported
Manojlovic, Vancouver Police Department	(2019)	Report and Resolutions	Law Enforcement	Police data	N/A	Bitcoin	Income tax scam (phishing style but referred to as above), other scams	Not reported
Nelson, Congressional Research Service	(2019)	Testimony	Legislative	Other studies	N/A	N/A (lots of cryptocurrencies discussed but not in context of fraud)	ICO scams	Not reported
NHS National Services Scotland	(2020)	Intelligence Alert	Healthcare	Anecdotal evidence	N/A	Bitcoin	COVID-related scams, cryptojacking, high yield investment programme, phishing, malware	Partially reported
Scott, Congressional Research Service	(2020)	Report	Legislative	Court cases	N/A	Bitcoin	Unfair and deceptive acts, unspecified fraud	Not reported
Securities and Exchange Commission Philippines	(2020)	Alert	Financial Services	Anecdotal evidence	N/A	N/A	Ponzi scheme	Fully reported

## Appendix 3

Definitions denoted with an asterisk (*), indicate types of fraud newly identified in the November 2020 update of the academic literature review. This information can also be found at https://osf.io/7w9mu/?view_only=c9ad3a1e2ed54dae9b1a0fc2807f144f. See Tables 6, 7 and 8.

**Table 6:** Description of fraud types identified in the academic literature

Label	Description
Ponzi scheme/ high yield investment programme	Ponzi schemes and high yield investment programmes are the cryptocurrency version of Charles Ponzi’s scam technique, where outlandish interest rates are promised in return for investments. Returns on these investments are paid to investors with funds invested by new users that join the scheme until it is no longer possible to find new victims (Bartoletti, et al., 2018; Baum, 2018; Pryzmont, 2016; Reddy et al., 2018; Vasek, 2017; Vasek et al., 2015)
Initial Coin Offering (ICO) scams	ICOs involve fundraising, often crowdfunding, to launch a new cryptocurrency (Anderson et al., 2019). Fraudulent ICOs, on the other hand, lure investors into paying money into cryptocurrencies for simple theft, or as part of pump-and-dump and Ponzi schemes (Barnes, 2018; Baum, 2018)
Phishing*	Phishing involves creating a fake version of an official website (or email, etc. and, in this case, cryptocurrency websites) and getting users to input their private information on this website (Chen et al., 2020a)
Pump-and-dump schemes	Pump-and-dump schemes are a type of stock market fraud and have been committed since the 1700s. They have recently been applied to cryptocurrencies. In the context of cryptocurrencies, fraudsters accumulate volumes of a low-value currency and then aim to artificially inflate its price by spreading misinformation, typically as a coordinated effort over the internet. When the value of the cryptocurrency increases, they sell everything to make a profit (Barnes, 2018; Baum, 2018; Chen et al., 2019c)
Market manipulation	Market manipulation refers to market participants (including exchanges) and bots attempting to change the price of a cryptocurrency (ur Rehman et al., 2020)
Exchange scam	Scams related to cryptocurrency exchange platforms entail the purposeful closing of a platform leading to financial losses for the cryptocurrency owners (Samsudeen et al., 2019). To that end, fraudulent exchange services entice victims through unique payment features or high exchange rates (Vasek, 2017; Vasek et al., 2015). Once victims have bought a cryptocurrency, the scammers simply close the exchange, taking the victims’ money without any repayment
Scam wallet	Scam wallets are fraudulent services that masquerade as cryptocurrency wallets to siphon some or all of the currency transferred to them (Vasek, 2017; Vasek et al., 2015)
Smart contracts honeypots	Smart contracts honeypots are smart contracts that seemingly contain design flaws. Users (the victims of this fraud) attempt to exploit these flaws, only to find that this perceived vulnerability did not exist. Instead, the code of the contract, when executed, does things like freeze their funds and only make them accessible to the scammer. For example, the honeypot could be set up to (appear to) leak funds (the bait) which a user may want to exploit by fulfilling the contract (e.g., paying a defined amount of cryptocurrency). The trap is that the code of the contract does not actually leak any funds but freezes them (for a detailed review, see Torres et al., 2019)
Mining scam	Victims invest in cryptocurrency mining operations in the hope of getting larger sums back, only to never receive a pay-out (Vasek, 2017; Vasek et al., 2015)
Fraudulent cryptocoins	No definition was reported in the reviewed studies. However, Higgins (2017) defines this type of fraud as the unauthorised use of names from established companies to gain the trust of potential investors
Smart Ponzi Scheme	Smart Ponzi schemes apply the classic Ponzi schemes technique to smart contract platforms (Bartoletti et al., 2017; Chen et al., 2018a, d). The scammer makes money by taking parts of the investments of the victims for themselves rather than genuinely investing it. High interest rates or returns are paid with the investments of others rather than through a genuine increase in value
Mining malware / cryptojacking	Mining Malware, also called ‘cryptojacking’, refers to malware programmes that run on victims’ machines and exploit the CPU to mine cryptocurrencies on behalf of the criminal (Anderson et al., 2019)
Securities fraud*	Securities fraud is not defined in the literature (though specific references are made to the U.S. statutory definition). It involves carrying out a scheme to defraud in connection with a registered security or ‘to obtain, by means of false or fraudulent pretenses [*sic.*], representations, or promises, any money or property in connection with the purchase or sale of…any security’ (Corporate & Criminal Fraud Accountability Act of, 2002, 2009). In this context, said money or property could include crypto assets and the security itself could be a crypto asset
Identity theft*	No definition of identity theft was included in the literature reviewed. However, the U.S. federal statutory definition is as follows: someone who ‘knowingly transfers or uses, without lawful authority, a means of identification of another person with the intent to commit, or to aid and abet, any unlawful activity that constitutes a violation of Federal law, or that constitutes a felony under any applicable State or local law’ (Identity Theft & Assumption Deterrence Act of, 1998, 2006)
Wire fraud*	No definition of wire fraud was reported in the literature, though it is understood to refer to the U.S. statutory definition of the same. According to the U.S. Code, wire fraud involves ‘any scheme or artifice to defraud, or for obtaining money or property by means of false or fraudulent pretenses [*sic.*], representations, or promises, transmits or causes to be transmitted by means of wire…in interstate or foreign commerce, any writings, signs, signals, pictures, or sounds for the purpose of executing such scheme or artifice’(18 U.S.C. § 1343)
Wash trading*	Wash trading was not defined in the literature. However, the UK Financial Conduct Authority defines it in the Market Abuse Regulation as ‘a sale or purchase of a qualifying investment where there is no change in beneficial interest or market risk, or where the transfer or beneficial interest or market risk is only between parties acting in concert or collusion, other than for legitimate reasons’ (Financial Conduct Authority, 2021). In this case, the sale or purchase could be completed using cryptocurrencies or the qualifying investment itself could be a crypto asset
Selfish mining*	Though selfish mining is not prohibited, per se, one article refers to it specifically as fraud (Phan et al., 2019). Selfish mining involves miners purposefully hiding blocks they have found so they can secretly mine on top of them, causing other miners to waste their computing power in trying to mine a block that has already been found. This allows the selfish miner to fork the blockchain, essentially enabling miners to carry out a 51% attack, but with a far smaller proportion of the overall hashing power (as little as 25%) (Phan et al., 2019)
Romance scams*	Romance scams involve a nefarious actor gaining an individual’s trust by engaging in a romantic relationship with them. Once they have received said trust, the perpetrator requests money (in this case, cryptocurrency) from the victim (usually for something like an urgent surgery, because they temporarily cannot access their bank, etc.) (Navarro, 2019)
Pyramid schemes*	In a pyramid scheme, participants earn money by recruiting other members to the scheme (in this context, a cryptocurrency investment scheme), rather than by delivering investments, products, or services (Jiaying, 2020)
Malware scams*	Malware prohibits victims’ access to their phones or computers until they pay a ransom in cryptocurrency (Xia et al., 2020a). Traditionally, this type of scam is more specifically referred to as ‘ransomware’, a type of malware
Insider trading*	The definition of insider trading was not reported in the literature reviewed. However, the U.S. Securities and Exchange Commission defines it as ‘buying or selling a security, in breach of a fiduciary duty or other relationship of trust and confidence, on the basis of material, nonpublic [*sic*.] information about the security’ (U.S. Securities and Exchange Commission, n.d.). To be considered crypto fraud, such a security would need to be a crypto asset
Imposter websites / apps*	This type of fraud was not defined in the literature. From the context of the article in which it appears, this form of fraud was understood to refer to creating fake versions of an official website or app (such as an exchange app, etc.) (Scheau et al., 2020)^a^
Giveaway scams*	In a giveaway scam, a fraudster promises to give victims a reward for sending him/her a particular amount of cryptocurrency (which is never ultimately delivered) (Xia et al., 2020a)
Fake agencies	Scammers pretend to be an existing exchange or government organization to steal cryptocurrency from customers (Samsudeen et al., 2019)
Donation scams*	In a donation scam, a fraudster will pretend to be from a public organisation purporting to raise money (using cryptocurrency) for a worthy cause that does not actually exist (Xia et al., 2020a)
Blackmail scams*	Blackmail scams were defined and discussed in the studies in the context of COVID-19. They refer to individuals claiming they will spread coronavirus unless the victim sends them cryptocurrency (Xia et al., 2020a)
Arbitrage scams*	Arbitrage refers to investors profiting off price imbalances in the market. Scammers often combine arbitrage with counterfeit cryptocoins, i.e., they provide a scam address for the victim to send cryptocurrency to (to take advantage of an arbitrage opportunity). Rather than returning their profits, they send only counterfeit tokens to the victim (Gao et al., 2020)
Airdrop scams*	Scammers promise to give various victims a free cryptocurrency token. Rather than providing the real cryptocurrency, they often send victims counterfeit tokens (Gao et al., 2020). In other cases, they airdrop token to trick a user into approving access to their online wallet; the scammer subsequently drains funds from their wallet
Advance-fee scam*	An advance-fee scam involves convincing a victim to send cryptocurrency to a particular address. The scammer promises to return the full amount and more (though this money never arrives) (Phillips & Wilder, 2020)

^a^Notably, this definition is similar, if not nearly identical, to that of phishing. However, since the literature defined it as a unique type of fraud, it is included here as such.

**Table 7:** Description of the fraud types identified in the private sector literature

Label	Description
SIM swapping	SIM swapping refers to fraudsters moving their victim’s phone number to a SIM card they control. Getting access to the victim’s phone number enables attackers to break into their accounts (such as cryptocurrency exchange accounts) (CipherTrace, 2018)
Commodity fraud	The definition of commodity fraud was not reported in the private sector literature. The U.S. Code defines it as carrying out a scheme ‘to defraud any person in connection with any commodity for future delivery, or any option on a commodity for future delivery’ or ‘to obtain, by means of false or fraudulent pretenses [*sic.*], representations, or promises, any money or property in connection with the purchase or sale of any commodity for future delivery, or any option on a commodity for future delivery’ (Corporate & Criminal Fraud Accountability Act of, 2002, 2009). The commodity in this case would be a crypto asset
Access device fraud	The private sector literature failed to report the definition of access device fraud. U.S. statute defines a perpetrator thereof as someone who ‘knowingly and with intent to defraud produces, uses, or traffics in one or more’ of items like ‘counterfeit access devices’; ‘unauthorized access devices’; ‘a telecommunications instrument that has been modified or altered to obtain unauthorized use of telecommunications services’; or ‘a scanning receiver’; among others (Counterfeit Access Device and Computer Fraud and Abuse Act of 1984, 2015)
CPO / CTA fraud	The definition of CPO / CTA fraud was not reported in the private sector literature. We understand this to refer to fraud committed by Commodity Pool Operators or Commodity Trading Advisors, in this case, fraud involving cryptocurrency (Ropes & Gray, 2019)
Credential stuffing	Credential stuffing occurs when brute-force attackers automatically try huge sets of login credentials in login pages in an attempt to access an account which exists (Krone et al., 2018). Attackers could use credential stuffing to access custodial wallet or exchange accounts^a^
Discount scams	The private sector literature failed to define discount scams. From the context in which they were mentioned, they appear to involve the promise of discounts for early investors in a cryptocurrency. The purported discounts may be for a counterfeit or fraudulent cryptocurrency (Bolster, 2019)
Dusting	Dusting involves scammers sending small amount of cryptocurrency to lots of addresses. The fraudster can then track these funds in an attempt to figure out which addresses are housed in the same wallet or identify wallet holders. They then use this information for targeted phishing or blackmail scams (Musiala et al., 2020). It is worth noting that dusting is not necessarily malicious; it has also been used for advertisement purposes
Embezzlement	Embezzlement was not defined in the private sector literature. However, the U.S. Supreme Court defined embezzlement ‘the fraudulent appropriation of property by a person to whom such property has been entrusted, or into whose hands it has lawfully come’ (Moore v. United States, 1895). In the context of cryptocurrency fraud, this would mean the misappropriation of crypto assets
Forex fraud	The private sector literature did not include a definition of forex fraud. The Financial Conduct Authority states that these scams involve unauthorised foreign exchange trading and brokerage firms who ‘promise very high returns and guaranteed profits’ (Financial Conduct Authority, 2020), in this case, from exchanging cryptocurrencies. Generally, victims will initially receive some returns, but, following further investment, the scam forex firm will halt all communication (Financial Conduct Authority, 2020)
Issuing false account statements in connection with soliciting investments	This was not defined in the literature, however, from the context in which it was mentioned, appears to refer to scammers publicising fake profit and loss information from their cryptocurrency ‘investment opportunity’ in order to entice new investors to join their scam (Malyshev et al., 2018)
Options fraud	Options fraud was not defined in the private sector literature. This review, again, interpreted the literature as referring to the U.S. statutory definition, namely, ‘to defraud any person in connection with…any option on a commodity for future delivery’ or ‘to obtain, by means of false or fraudulent pretenses [*sic.*], representations, or promises, any money or property in connection with the purchase or sale of…any option on a commodity for future delivery’ (in this context, an option on a crypto asset) (Corporate & Criminal Fraud Accountability Act of, 2002, 2009)
Impersonating celebrities or a federal employee	The private sector literature did not define scams involving the impersonation of a federal employee (Lucking & Aravind, 2019). The public sector literature specifically referred to impersonation scams involving celebrities, defining them as involving a scammer using ‘the image, name, and personal characteristics of a well-known person to sell a product or service’ (Australian Competition & Consumer Commission, 2020). This could involve, for example, a fraudster impersonating a celebrity to recruit victims to a cryptocurrency investment scam. We understand the definition of impersonating a federal employee to be largely the same (albeit with the perpetrator impersonating different targets). In practice, this is closely related to giveaway and advance-fee frauds^b^
Exploiting vulnerabilities	Though this was not defined in the publication that mentioned it, exploiting vulnerabilities is understood to refer to any fraudulent behaviour enabled by web browser, software, hardware or firmware security issues (PYMNTS.com & Trulioo, 2019). This could also potentially apply to exploiting vulnerabilities in smart contracts, though this was unclear from the context in which it was discussed
Ransomware	The private sector literature that referred to ransomware specifically as a type of fraud defined it as malware that controls a victim’s computer or device and ‘holds it hostage until the victim pays the hackers to regain access’ (Musiala et al., 2020). Generally, the hackers require payment in cryptocurrency

^a^In contrast, in the academic literature, this was specifically defined as phishing (Navarro, 2019). While the source of these sets of login credentials may be phishing, the definition provided above is more accurate.

^b^This is similar to the definition of ‘fake agencies’, as defined in the academic literature.

**Table 8:** Description of fraud types identified in the public sector literature

Label	Description
COVID-related scams	The publication that referred to COVID-related cryptocurrency scams did not provide a definition thereof. However, it is implied that this refers to any scam related to COVID-19 that requires payment in cryptocurrencies, such as donation scams, payment for fake personal protective equipment, etc. (NHS National Services Scotland, 2020). We note that Xia et al. (2020a) also define various scams in the context of COVID, which we have included separately above (see, for example, definitions of malware scams, giveaway scams, donation scams, and blackmail scams)
False signals of supply and demand (wash trading, layering, spoofing)	False signals of supply and demand were not defined in the public sector literature. The UK Financial Conduct Authority, in the Market Abuse Regulations, defines this as providing information ‘which is likely to give the regular user a false or misleading impression as to the supply of, or the demand for, or the price or value of a qualifying investment or relevant product’, for example, of a crypto asset (Financial Conduct Authority, 2021)
Income tax scams	The public sector publication referring to income tax scams did not offer a definition thereof (Manojlovic, 2019). However, in describing the scam, it appears to involve someone impersonating federal employees (defined above), specifically, in this case, Canada Revenue Agency employees (Manojlovic, 2019)
Unfair and deceptive acts	This was not defined in the literature. However, it is understood to refer to the Federal Trade Commission Act, which prohibits any practice that ‘causes or is likely to cause substantial injury to consumers; cannot be reasonably avoided by consumers; and is not outweighed by countervailing benefits to consumers or to competition’ or ‘where a representation, omission, or practice misleads or is likely to mislead the consumer; a consumer’s interpretation of the representation, omission, or practice is considered reasonable under the circumstances; and the misleading representation, omission, or practice is material’ (Federal Trade Commission Act, 2012)

## Abbreviations

PRISMA-ScR: Preferred Reporting Items for Systematic reviews and Meta-Analyses extension for Scoping Reviews
PoW: Proof of Work
GS: Google Scholar
HYIPs: High yield investment programmes
ICOs: Initial coin offerings
CPO: Commodity Pool Operator
CTA: Commodity Trading Advisor

## Footnotes

1 Not all cryptocurrencies employ PoW. Other mechanisms, such as Proof of Stake, are used by other cryptocurrencies.

2 In designing the scoping review, we conducted test searches of multiple databases using various search strings with varying levels of specificity. Ultimately, Google Scholar provided the most comprehensive results, while reducing irrelevant noise in our results. In contrast to other publication aggregators, such as the Web of Science, GS is faster in indexing published work, especially from pre-print servers (i.e., where researchers make publications available without a paywall or before publication in conference proceedings or a journal). To map existing research on cryptocurrency fraud, this review required academic search engines and databases with broad coverage. Previous studies suggested that GS provides the best scope among the available databases. For instance, Gusenbauer (2019) compared the coverage of 12 databases and found GS to provide the most comprehensive range of academic publications. Martín-Martín, et al. (2018) analysed GS, Scopus, and Web of Science concluding that GS identified the largest proportion of citations across a broad spectrum of subject areas. Against this background, we tested our search on GS, ProQuest, Scopus, and Web of Science in April 2019. All of these databases, including GS, include results behind paywalls as well as open access sources. The results suggested GS was the only database with comprehensive coverage of academic publications. Given these findings, we selected GS as the information source for this scoping review on cryptocurrency fraud.

3 Note that Google Scholar does not support the wildcard function. However, from our test searches, using wildcard characters primarily resulted in more noise, rather than better findings.

4 Ineligible publication types included agendas marketing materials, blog posts, indices, infographics, statutes, magazines, reading lists, contracts, CVs, course catalogues, court cases, correspondence, op eds, press releases, news articles, websites, newsletters, and PowerPoints.

5 Definitions of all types of fraud identified in this scoping review can also be found at https://osf.io/7w9mu/?view_only=c9ad3a1e2ed54dae9b1a0fc2807f144f.

6 For a full list of definitions identified in this scoping review, see https://osf.io/7w9mu/?view_only=c9ad3a1e2ed54dae9b1a0fc2807f144f.

7 See also https://osf.io/7w9mu/?view_only=c9ad3a1e2ed54dae9b1a0fc2807f144f.

8 The full schedule of the event can be found at https://drive.google.com/file/d/1EqRYLUCTQ6Vn3oN_CQ0RSOa1Kgmpbq93/view.

9 We exclude here discussion of problems identified which specifically relate to money laundering or areas of crime other than fraud. Participants identified the following other crimes: money laundering using Bitcoin ATMs, cryptocurrency money mules, and cryptocurrency transaction extortion. More detail can be found in the following policy brief: https://www.ucl.ac.uk/jill-dando-institute/sites/jill-dando-institute/files/ucl_cryptocurrencies_and_future_crime_policy_briefing_feb2021_compressed_1.pdf.

10 The Mentimeter polling software allowed us to display the questions using the Mentimeter app interface (using an Internet connection). That interface was displayed on a big screen, and each participant could use their smartphone to obtain access to answer the questions. Once they provided an answer, their response was visible (in anonymised form) on the screen so that the participants could see their judgments in real-time.

11 For example, many standards have been developed for the Internet; perhaps the cryptocurrency arena could learn from this example. See, for example, the work of the Internet Engineering Task Force.

## References

[CR1] Advertising Standards Authority & Committees of Advertising Practice. (2019). *Using Technology for Good* [Annual Report]. https://www.asa.org.uk/uploads/assets/68dd32b5-ae6a-4993-820a3ff8f1163b8e/c02c8290-0ceb-4946-a98e383d5ee79796/ASA-CAP-2019-Annaul-Report-Full-Version-Singles.pdf

[CR2] Akin Gump Strauss Hauer & Feld LLP. (2018). *Federal judge adopts CFTC position that cryptocurrencies are commodities*. Akin Gump Strauss Hauer & Feld LLP. https://www.akingump.com/a/web/65522/aoiZE/litigation-alert-federal-judge-adopts-cftc-position-that-crypt.pdf

[CR3] Anderson, R., Barton, C., Rainer, B., Clayton, R., Ga, C., Grasso, T., Levi, M., Moore, T., & Vasek, M. (2019). Measuring the Changing Cost of Cybercrime Our Framework for Analysing the Costs of Cybercrime. *Workshop on the Economics of Information Security (WEIS)*, 1–32.

[CR4] Arksey H, O’Malley L (2005). Scoping studies: Towards a methodological framework. International Journal of Social Research Methodology: Theory and Practice.

[CR34] Australian Competition & Consumer Commission. (2018). *Targeting scams*. http://www.keepmeposted.org.au/wp-content/uploads/2018/05/f1240_targeting-scams-report.pdf

[CR5] Australian Competition and Consumer Commission. (2019). *Targeting scams. Report of the ACCC on scams activity 2018* (Issue May).

[CR37] Australian Competition & Consumer Commission. (2020). *Targeting scams 2019*. https://www.accc.gov.au/system/files/1657RPT_Targeting%20scams%202019_FA.pdf

[CR6] Badawi E, Jourdan G-V (2020). Cryptocurrencies emerging threats and defensive mechanisms: A systematic literature review. IEEE Access.

[CR7] Badawi E, Jourdan G-V, Bochmann G, Onut I-V (2020). An automatic detection and analysis of the bitcoin generator scam. IEEE European Symposium on Security and Privacy Workshops (EuroS&PW).

[CR8] Barnes, P. (2018). Crypto Currency and its Susceptibility to Speculative Bubbles, Manipulation, Scams and Fraud. *Journal of Advanced Studies in Finance*, *9*(2), 60. 10.14505//jasf.v9.2(18).03

[CR9] Bartoletti, M., Carta, S., Cimoli, T., & Saia, R. (2017). *Dissecting Ponzi schemes on Ethereum: Identification, analysis, and impact*.

[CR10] Bartoletti, M., Pes, B., & Serusi, S. (2018). Data mining for detecting bitcoin ponzi schemes. In *Proceedings—2018 Crypto Valley Conference on Blockchain Technology, CVCBT 2018*, pp. 75–84. 10.1109/CVCBT.2018.00014

[CR11] Bartoletti M, Carta S, Cimoli T, Saia R (2020). Dissecting Ponzi schemes on Ethereum: Identification, analysis, and impact. Future Generation Computer Systems.

[CR12] Baum, S. (2018). *Cryptocurrency fraud: A look into the frontier of fraud*.

[CR13] Bolster. (2019). *State of Phishing & Online Counterfeiting* [Annual Report]. https://bolster.ai/assets/files/reports/2019.pdf

[CR14] Boshmaf, Y., Al Jawaheri, H., & Al Sabah, M. (2019). BlockTag: Design and applications of a tagging system for blockchain analysis. In *IFIP International Conference on ICT Systems Security and Privacy Protection*, pp. 299–313.

[CR15] Boshmaf, Y., Elvitigala, C., Al Jawaheri, H., Wijesekera, P., & Al Sabah, M. (2020). Investigating MMM Ponzi scheme on Bitcoin. In *Proceedings of the 15th ACM Asia Conference on Computer and Communications Security*, pp. 519–530.

[CR16] Burrus, J. (2018). *Fighting financial crime in the age of cryptocurrencies* (p. 3). Refinitiv. https://www.refinitiv.com/content/dam/marketing/en_us/documents/expert-talks/world-check-expert-talk-fighting-financial-crime.pdf

[CR17] Capobianco, A. (2019). *Digital Disruption in Financial Markets—Note by the United States* (p. 10). Organisation for Economic Co-operation and Development. https://www.justice.gov/atr/page/file/1313821/download

[CR18] Chainalysis. (2019). *Crypto Crime Report*. Chainalysis. https://uploads-ssl.webflow.com/5a9360f88433cb00018022c2/5c4f67ee7deb5948e2941fda_Chainalysis%20January%202019%20Crypto%20Crime%20Report.pdf

[CR19] Chainalysis. (2020). *The 2020 Geography of Cryptocurrency Report*. https://go.chainalysis.com/rs/503-FAP-074/images/2020-Geography-of-Crypto.pdf

[CR20] Chainalysis. (2021, January 19). Crypto Crime Summarized: Scams and Darknet Markets Dominated 2020 by Revenue, But Ransomware Is the Bigger Story. *Chainalysis Insights*. https://blog.chainalysis.com/reports/2021-crypto-crime-report-intro-ransomware-scams-darknet-markets

[CR24] Chen, W., Guo, X., Chen, Z., Zheng, Z., & Lu, Y. (2020). Phishing Scam Detection on Ethereum: Towards Financial Security for Blockchain Ecosystem. *Proceedings of the Twenty-Ninth International Joint Conference on Artificial Intelligence Special Track on AI in FinTech.*, 4506–4512.

[CR25] Chen W, Guo X, Chen Z, Zheng Z, Lu Y, Li Y (2020). Honeypot contract risk warning on ethereum smart contracts. IEEE International Conference on Joint Cloud Computing.

[CR22] Chen, W., Wu, J., Zheng, Z., Chen, C., & Zhou, Y. (2019a). Market Manipulation of Bitcoin: Evidence from Mining the Mt. Gox Transaction Network. In *Proceedings—IEEE INFOCOM*, *2019-April* (November 2013), pp. 964–972. 10.1109/INFOCOM.2019.8737364

[CR23] Chen, W., Xu, Y., Zheng, Z., Zhou, Y., Yang, J. E., & Bian, J. (2019b). Detecting ‘Pump & dump schemes’ on cryptocurrency market using an improved apriori algorithm. In *Proceedings—13th IEEE International Conference on Service-Oriented System Engineering, SOSE 2019, 10th International Workshop on Joint Cloud Computing, JCC 2019 and 2019 IEEE International Workshop on Cloud Computing in Robotic Systems, CCRS 2019*, pp. 293–298. 10.1109/SOSE.2019.00050

[CR26] Chen W, Xu Y, Zheng Z, Zhou Y, Yang JE, Bian J (2019). Detecting" Pump & Dump Schemes" on cryptocurrency market using an improved Apriori Algorithm. IEEE International Conference on Service-Oriented System Engineering (SOSE).

[CR21] Chen, W., Zheng, Z., Cui, J., Ngai, E., Zheng, P., & Zhou, Y. (2018). *Detecting Ponzi Schemes on Ethereum*. pp. 1409–1418. 10.1145/3178876.3186046

[CR27] Chen W, Zheng Z, Ngai ECH, Zheng P, Zhou Y (2019). Exploiting blockchain data to detect smart ponzi schemes on ethereum. IEEE Access.

[CR28] Chen Z, Khoa L, Teoh E, Nazir A, Karuppiah E, Lam K (2018). Machine learning techniques for anti-money laundering (AML) solutions in suspicious transaction detection: A review. Knowledge and Information Systems.

[CR29] Chernin, A., Moran, N., & Mola, S. (2020). *Trends in CFTC Virtual Currency Enforcement Actions: 2015-Q2 2020* (p. 14). Cornerstone Research. https://www.cornerstone.com/Publications/Reports/Trends-in-CFTC-Virtual-Currency-Enforcement-Actions-2015-Q2-2020/Trends-in-CFTC-Virtual-Currency-Enforcement-Actions-2015-Q2-2020.pdf

[CR30] CipherTrace. (2018). *Cryptocurrency Anti-Money Laundering Report* (p. 22). https://ciphertrace.com/crypto-aml-report-2018q3.pdf

[CR31] CipherTrace. (2020). *ALERT: Malicious Crypto Browser Extension—Masked MetaMask*. https://ciphertrace.com/alert-malicious-crypto-browser-extension-masked-metamask/

[CR32] Cohen, D. O., & Crance, R. (2018). *Taking a trip around the regulatory block: U.S. regulation of blockchain and digital assets* (Financial Services Litigation Alert, p. 4). Schnader Harrison Segal & Lewis LLP. https://www.schnader.com/wp-content/uploads/2019/02/ALERT-Financial-Services-Blockchain-and-Cryptocurrency-Regulation-7-31-18.pdf

[CR33] CoinMarketCap. (2021). *Today’s cryptocurrency prices by market cap*. CoinMarketCap. https://coinmarketcap.com/

[CR36] Conley, B., Echert, J., Fuller, A., Lewis, H., & Lunday, C. (2015). Cryptocurrencies: An introduction for policy makers. *Technology Law and Public Policy Clinic*.

[CR38] Conti M, Gangwal A, Ruj S (2018). On the economic significance of ransomware campaigns: A Bitcoin transactions perspective. Computers & Security.

[CR39] Conway, L. (2021). *Bitcoin Halving*. Investopedia. https://www.investopedia.com/bitcoin-halving-4843769

[CR40] Corporate and Criminal Fraud Accountability Act of 2002, 18 United States Code § 1348 (2009). https://www.law.cornell.edu/uscode/text/18/1348

[CR41] Coulter, B. B. (2018). *CFTC Can Regulate Cryptocurrencies as Commodities* (Burr Alert, p. 2). Burr Forman LLP. https://www.burr.com/wp-content/uploads/2018/03/ALERT_CFTC-Can-Regulate-Cryptocurrencies-as-Commodities_Coulter.pdf

[CR42] Global Blockchain Business Council. (2020). *Chain Reaction: Blockchain Enters the Mainstream* [Annual Report]. Accenture. https://www.lw.com/thoughtLeadership/gbbc-report-blockchain-enters-mainstream

[CR43] Crowell & Moring. (2018). *Cryptocurrency in Small Bytes: The CFTC Chalks Up a Preliminary Win* [Client Alert]. https://www.crowell.com/NewsEvents/AlertsNewsletters/all/Cryptocurrency-in-Small-Bytes-The-CFTC-Chalks-Up-a-Preliminary-Win/pdf

[CR44] Crowley, B. J. (2018). *The Legal and Regulatory Issues Surrounding Cryptocurrency*.

[CR46] Counterfeit Access Device and Computer Fraud and Abuse Act of 1984, 18 United States Code § 1029 (2015). https://www.law.cornell.edu/uscode/text/18/1029

[CR45] Desai, V., Coderre, R., Kizzee, C., & Kalra, H. (2019). *Retail and Hospitality Threat Trend report* (No. 190147; p. 46). Accenture.

[CR47] Devonshires. (2018). *Litigation Know How: Staying Ahead of the Game*. Devonshires. https://www.devonshires.com/wp-content/uploads/2017/07/Com-Lit-Brief-October-18.pdf

[CR48] Dubois, S. M. (2019). *A Baseline Assessment of Law Enforcement Cryptocurrency Investigations.* University of Central Lancashire.

[CR49] Duff KB, Hays G (2018). Recent developments in the war on cryptocurrency fraud. The Receiver.

[CR50] Eastham EB (2020). Morrison and cryptocurrencies: Is it time to revisit the extraterritorial application of rule 10B–5. Ga. J. Int’l & Comp. l..

[CR51] Elder, R., & Rotunda, J. (2018). *Widespread Fraud Found in Cryptocurrency Offerings* (Investor Alert: Cryptocurrencies). Texas State Securities Board. https://www.ssb.texas.gov/sites/default/files/CRYPTO%20SWEEP%20report%2002112019%20UPDATE.pdf

[CR52] Elwell, C. K., Murphy, M. M., & Seitzinger, M. V. (2014). *Bitcoin: Questions, Answers, and Analysis of Legal Issues* (p. 20). Congressional Research Service.

[CR53] Eversheds Sutherland Ltd. (2018). *Navigating the issues Securities Enforcement Global Update*. https://us.eversheds-sutherland.com/mobile/portalresource/lookup/poid/Z1tOl9NPluKPtDNIqLMRV56Pab6TfzcRXncKbDtRr9tObDdEpW3CmS3!/fileUpload.name=/Securities-Enforcement-Global-Update_Fall-2018.pdf

[CR54] Federal Trade Commission Act, 15 United States Code § 45 (2012). https://www.law.cornell.edu/uscode/text/15/45

[CR55] Financial Integrity Network. (2018). *Virtual Currencies: Momentum Building For Regulation and Enforcement* [Policy Alert].

[CR56] Financial Conduct Authority. (2019). *Cryptoasset investment scams*. https://www.fca.org.uk/scamsmart/cryptoasset-investment-scams

[CR57] Financial Conduct Authority. (2020). *Forex trading scams*. https://www.fca.org.uk/scamsmart/forex-trading-scams

[CR58] Financial Conduct Authority. (2021). *FCA Handbook*. https://www.handbook.fca.org.uk/handbook/MAR/1/6.html?date=2016-03-07

[CR60] Frankel T (2012). The ponzi scheme puzzle: A history and analysis of con artists and victims. Oxford University Press.

[CR61] FSMA. (2020). *Cryptocurrency fraud: The FSMA updates its list of suspicious sites* (p. 1) [Warnings]. https://web3.cmvm.pt/SDI/IFs/app/docs/fsd852235.pdf

[CR62] Gandal N, Hamrick JT, Moore T, Oberman T (2018). Price manipulation in the Bitcoin ecosystem. Journal of Monetary Economics.

[CR63] Gao, B., Wang, H., Xia, P., Wu, S., Zhou, Y., Luo, X., & Tyson, G. (2020). Tracking Counterfeit Cryptocurrency End-to-end. *ArXiv Preprint * ArXiv:2011.02673.

[CR64] Garg, S. C., Sawhney, A. P., Tyagi, A., & Kanungo, B. P. (2019). *Report of the Committee to propose specific actions to be taken in relation to Virtual Currencies* (Committee on Virtual Currencies). Department of Economic Affairs, Ministry of Finance. https://dea.gov.in/sites/default/files/Approved%20and%20Signed%20Report%20and%20Bill%20of%20IMC%20on%20VCs%2028%20Feb%202019.pdf

[CR65] Goodlett, B. (2020). *Texas cracks down on cryptocurrency fraud: 2020 developments* (Accelerate, p. 2). DLA Piper.

[CR67] Gusenbauer, M. (2019). Google Scholar to overshadow them all? Comparing the sizes of 12 academic search engines and bibliographic databases. In *Scientometrics* (Vol. 118, Issue 1). Springer International Publishing. 10.1007/s11192-018-2958-5

[CR68] Halverson, G. (2020). *Global Payments 2020–30 A quantum shift in the next decade* [Submission to RBA Payments Boards]. McLean Roche Consulting Group. https://www.rba.gov.au/payments-and-infrastructure/submissions/review-of-retail-payments-regulation/mclean-roche-consulting.pdf

[CR69] Hays G (2018). Receiver keeping up with the cryptos. The Receiver.

[CR70] Hedrich, W., Chalmers, B., Viet, P. H., & Koh, J. (2018). *14 Shades of risk in Asia-Pacific*. Marsh & McLennan Companies.

[CR71] Henning, P. J. (2019). *A Taxonomy of Cryptocurrency Enforcement Actions* (SSRN Scholarly Paper ID 3483198). Social Science Research Network. https://papers.ssrn.com/abstract=3483198

[CR72] Higgins, S. (2017). A Digital Currency Scam is Misusing the Rothschild Family Name. *CoinDesk*. https://www.coindesk.com/rothschild-advisory-warns-fraudulent-digital-currency

[CR145] HM Treasury, Financial Conduct Authority, & Bank of England. (2018). *Cryptoassets Taskforce: Final report* (p. 58). https://assets.publishing.service.gov.uk/government/uploads/system/uploads/attachment_data/file/752070/cryptoassets_taskforce_final_report_final_web.pdf

[CR139] Identity Theft and Assumption Deterrence Act of 1998, Pub. L. No. 3007, 18 United States Code (2006). https://www.law.cornell.edu/uscode/text/18/1028

[CR73] Insikt Group. (2018). *Shifting Patterns in Internet Use Reveal Adaptable and Innovative North Korean Ruling Elite*. https://www.recordedfuture.com/north-korea-internet-usage/

[CR59] Internet Engineering Task Force. (n.d.). *Internet standards*. IETF. Retrieved 16 March 2021, from https://www.ietf.org/standards/

[CR74] Jiaying, J. (2020). Regulating Blockchain? A Retrospective Assessment of China’s Blockchain Policies and Regulations. *Tsinghua China L. Rev.*, *12*.

[CR75] Jung E, Le Tilly M, Gehani A, Ge Y (2019). Data mining-based ethereum fraud detection. IEEE International Conference on Blockchain (Blockchain).

[CR76] Kamps J, Kleinberg B (2018). To the moon: Defining and detecting cryptocurrency pump-and-dumps. Crime Science.

[CR77] Keller, P., Florian, M., & Böhme, R. (2021). Collaborative Deanonymization. ArXiv:2005.03535* [Cs]*. http://arxiv.org/abs/2005.03535

[CR78] Kethineni S, Cao Y (2019). The rise in popularity of cryptocurrency and associated criminal activity. International Criminal Justice Review.

[CR79] Krone, M. J., Lukes, E. M., & McKibbin, C. (2018). Tales from the Crypt: Cryptocurrency is Here—How will crime insurers respond? *The Fidelity Law Journal*, *XXIV*, 86.

[CR80] Lašas, K., Kasputytė, G., Užupytė, R., & Krilavičius, T. (2020). Fraudulent behaviour identification in ethereum blockchain. *CEUR Workshop Proceedings [Electronic Resource]: IVUS 2020, Information Society and University Studies, Kaunas, Lithuania, 23 April, 2020: Proceedings. Aachen: CEUR-WS, 2020, Vol. 2698*.

[CR81] Law, J., & Martin, E. A. (2009). Fraud. In J. Law & E. A. Martin (Eds.), *Oxford dictionary of law* (7th ed.). Oxford.

[CR83] Leuz, C. (2018). *Regulatory approaches to combat retail investor fraud*. SEC Investor Advisory Committee. https://www.sec.gov/spotlight/investor-advisory-committee-2012/iac030818-leuz-remarks.pdf

[CR84] Levac D, Colquhoun H, O’Brien KK (2010). Scoping studies: Advancing the methodology. Implementation Science.

[CR85] LIFARS. (2018). *Bitcoin & cryptocurrency investment prospects & hacking incidents*. https://lifars.com/wp-content/uploads/2018/04/V2-Bitcoin-and-Cryptocurrency-Investment-Prospects-Hacking-Incidents.pdf

[CR86] Lim B, Murphy GE, Heda K, Hannotin C, Krauß O, Popp F, Maximenko A, Klutchareva E, Low C (2019). Blockchain 2018 year-in-review.

[CR87] Liu, X. F., Jiang, X.-J., Liu, S.-H., & Tse, C. K. (2020). Knowledge discovery in cryptocurrency transactions: A survey. *ArXiv Preprint * ArXiv:2010.01031.

[CR88] Lockhart J, Nuesser A (2018). Taking Caution: Financial Consumersand the Cryptoasset Sector (Investor Office.

[CR89] Lucking, D., & Aravind, V. (2019). Cryptocurrency as a Commodity: The CFTC’s Regulatory Framework. *Global Legal Insights*.

[CR90] Mabille, C. (2020). *Is cryptocurrency a store of value for the current crisis?* Finatic. https://www.finatic.be/bitcoin.pdf

[CR91] Malta Financial Services Authority. (2019). *MSFA Warning—Bitcoin Future*. https://web3.cmvm.pt/SDI/IFs/app/docs/fsd816904.pdf

[CR92] Malyshev P, Achilles J, Ottenberg J, Stumacher J (2018). CFTC enforcement trends in 2017 and considerations for 2018. Journal of Investment Compliance.

[CR93] Manojlovic, D. (2019). *Report to the Vancouver Police Board* (No. 1902P03). https://vancouver.ca/police/policeboard/agenda/2019/0221/1902P04-Proposed-CAPG-Resolutions.pdf?TB_iframe=true&width=370.8&height=658.8

[CR94] Martín-Martín A, Orduna-Malea E, Thelwall M, Delgado López-Cózar E (2018). Google Scholar, Web of Science, and Scopus: A systematic comparison of citations in 252 subject categories. Journal of Informetrics.

[CR95] McAvoy, D., Queenin, C., & Becker, B. (2018). *Court confirms CFTC jurisdiction over cryptocurrency fraud and that virtual currencies are commodities* (Now + Next, p. 2). Nixon Peabody LLP. https://www.nixonpeabody.com/-/media/Files/Alerts/2018-March/Court-confirms-CFTC-jurisdiction-over-cryptocurrency-fraud.ashx?la=en

[CR96] McGuire, M., & Dowling, S. (2013). Cyber crime: A review of the evidence. Research Report 75. Chapter 1: Cyber-dependent crimes. In *Home Office Research Report 75*.

[CR97] McGuire, M. (2019). *Social Media Platforms and the Cybercrime Economy* (Into the Web of Profit). Bromium. https://www.bromium.com/wp-content/uploads/2019/02/Bromium-Web-of-Profit-Social-Platforms-Report.pdf

[CR98] Meiklejohn S, Pomarole M, Jordan G, Levchenko K, McCoy D, Voelker GM, Savage S (2016). A fistful of Bitcoins: Characterizing payments among men with no names. Communications of the ACM.

[CR99] Moher D, Liberati A, Tetzlaff J, Altman DG (2009). Preferred reporting items for systematic reviews and meta-analyses: The PRISMA Statement. PLoS Medicine.

[CR100] Moore V. United States, (U.S. Supreme Court 1895).

[CR101] Möser M, Bohme R, Breuker D (2013). An inquiry into money laundering tools in the Bitcoin ecosystem. APWG ECrime Researchers Summit.

[CR102] Munn Z, Peters MDJ, Stern C, Tufanaru C, McArthur A, Aromataris E (2018). Systematic review or scoping review? Guidance for authors when choosing between a systematic or scoping review approach. BMC Medical Research Methodology.

[CR103] Murko, A., & Vrhovec, S. L. (2019). Bitcoin adoption: Scams and anonymity may not matter but trust into Bitcoin security does. In *Proceedings of the Third Central European Cybersecurity Conference*, pp. 1–6.

[CR104] Murphy, E. V., Murphy, M. M., & Seitzinger, M. V. (2015). *Bitcoin: Questions, answers, and analysis of legal issues*.

[CR105] Musiala, R. A. Jr., Goody, T. M., Reynolds, V., Tenery, L., McGrath, M., Rowland, C., & Sekhri, S. (2020). *Cryptocurrencies: Forensic techniques to meet the challenge of new fraud and corruption risks | FVS Eye on Fraud*. AICPA. https://future.aicpa.org/resources/download/cryptocurrencies-forensic-techniques-to-face-new-fraud-and-corruption-risks

[CR106] Najmy, W. G. (2019). Enhanced Tax Compliance for Cryptoasset Transactions. *Available at SSRN 3499659*.

[CR107] Nakamoto, S. (2008). *Bitcoin: A Peer-to-Peer Electronic Cash System*. 9.

[CR108] Narayanan, A., Bonneau, J., Felten, E., Miller, A., & Goldfeder, S. (2016). *Bitcoin and Cryptocurrency Technologies*.

[CR109] Navarro, R. R. (2019). *Preventative fraud measures for cryptocurrency exchanges: Mitigating the risk of cryptocurrency scams* [M.S., Utica College]. http://search.proquest.com/docview/2312801484/abstract/7B22A14839754F47PQ/1

[CR111] Nelson, R. M. (2019). *Examining regulatory frameworks for digital currencies and blockchain* (Testimony No. 7–5700; p. 15). Congressional Research Service. https://www.banking.senate.gov/imo/media/doc/Nelson%20Testimony%207-30-19.pdf

[CR112] Ngai, K. (2014). *Regulating cryptocurrency to prevent fraud and money laundering*.

[CR113] NHS National Services Scotland. (2020). *NHS counter fraud services rolling COVID-19 intelligence alert no. 14*. https://www.wihb.scot.nhs.uk/wp-content/uploads/2020/08/2020-08-03-CFS-Rolling-COVID-19-Intel-Alert-No14-Final.pdf

[CR114] Nilsen, A. I. (2019). *Limelight: Real-time detection of pump-and-dump events on cryptocurrency exchanges using deep learning*. UiT Norges arktiske universitet.

[CR115] Paré G, Trudel MC, Jaana M, Kitsiou S (2015). Synthesizing information systems knowledge: A typology of literature reviews. Information and Management.

[CR116] Parisi, D., Goldman, G., Baris, J. G., Nallengara, L., Sahni, R. A., Greene, N., Szaja, P. J., Reynolds, B., Anderson, S. W., & Oosterbaan, J. (2018). *Regulators and courts clarify virtual currency regulation, but overall framework remains murky*. Shearman & Sterling. http://www.legalexecutiveinstitute.com/wp-content/uploads/2018/05/345PM-Regulators.pdf

[CR117] Peters MDJ, Godfrey CM, Khalil H, McInerney P, Parker D, Soares CB (2015). Guidance for conducting systematic scoping reviews. International Journal of Evidence-Based Healthcare.

[CR118] Peterson J, Pearce PF, Ferguson LA, Langford CA (2017). Understanding scoping reviews: Definition, purpose, and process. Journal of the American Association of Nurse Practitioners.

[CR119] Pham MT, Rajić A, Greig JD, Sargeant JM, Papadopoulos A, Mcewen SA (2014). A scoping review of scoping reviews: Advancing the approach and enhancing the consistency. Research Synthesis Methods.

[CR120] Phan, L., Li, S., & Mentzer, K. (2019). Blockchain technology and the current discussion on fraud. *Computer Information Systems Journal*.

[CR121] Phillips, R., & Wilder, H. (2020). Tracing cryptocurrency scams: Clustering replicated advance-fee and Phishing Websites. *ArXiv Preprint * ArXiv:2005.14440.

[CR122] Podgor, E. S. (2019). Cryptocurrencies and securities fraud: In need of legal guidance. *Available at SSRN 3413384*.

[CR123] Pryzmont, P. (2016). *An empirical study of how Bitcoin related incidents impact its price volatility*. *August*, 62.

[CR124] PYMNTS.com & Trulioo. (2019). *Keeping crime out of crypto* (AML/KYC Tracker). https://www.pymnts.com/wp-content/uploads/2019/07/AML-KYC-Tracker-July-2019.pdf

[CR125] Reddy E, Minaar A (2018). Cryptocurrency: A tool and target for cybercrime. Acta Criminologica: Southern African Journal of Criminology.

[CR126] Reik, T. (2019). *Bitcoin Revisited*. Sprott Asset Management USA, Inc. https://www.sprottusa.com/media/2497/sprott-gold-report-bitcoin-revisited.pdf

[CR127] Rochemont, S. (2020). *A Cashless Society in 2019*.

[CR128] Rognone L, Hyde S, Zhang SS (2020). News sentiment in the cryptocurrency market: An empirical comparison with Forex. International Review of Financial Analysis.

[CR66] Ropes & Gray. (2019). *CFTC amends regulations applicable to asset managers including excluded and exempt CPOs and CTAs; action may be required*. http://www.ropesgray.com/en/newsroom/alerts/2019/12/CFTC-Amends-Regulations-Applicable-to-Asset-Managers-Including-Excluded-and-Exempt-CPOs-and-CTAs

[CR129] Samsudeen Z, Perera D, Fernando M (2019). Behavioral analysis of bitcoin users on illegal transactions. Advances in Science, Technology and Engineering Systems Journal.

[CR130] Schär, F. (2021). *Decentralized finance: On blockchain- and smart contract-based financial markets*. Federal Reserve Bank of St. Louis. https://research.stlouisfed.org/publications/review/2021/02/05/decentralized-finance-on-blockchain-and-smart-contract-based-financial-markets

[CR131] Scheau, M. C., Link to external site, this link will open in a new window, Crăciunescu, S. L., Brici, I., & Achim, M. V. (2020). A cryptocurrency spectrum short analysis. *Journal of Risk and Financial Management; Basel*, *13*(8), 184. 10.3390/jrfm13080184

[CR132] Scott, A. P. (2020). *Fintech: Overview of Financial Regulators and Recent Policy Approaches* (No. R46333; p. 38). Congressional Research Service. https://crsreports.congress.gov/product/pdf/R/R46333

[CR133] Securities and Exchange Commission. (2013). Ponzi schemes using virtual currencies. *SEC Pub. No. 153 (7/13)*.

[CR134] Securities and Exchange Comimssion Philippines. (2020). *SEC Advisory*. https://www.sec.gov.ph/wp-content/uploads/2020/10/2020Advisory_BITCOIN-DIGITAL.pdf

[CR135] Semenihin, A., & Kondrashin, A. (2018). *Leading role of state as a regulator of crypto currency*. *217*(ICSEAL), 329–334. 10.2991/icseal-18.2018.47

[CR136] Štefanko, L. (2018). *Cryptocurrency scams on Android* (ESET Research Whitepapers, p. 15). ESET. https://www.welivesecurity.com/wp-content/uploads/2018/02/Cryptocurrency_Scams_on_Android.pdf

[CR137] Shearman & Sterling. (2018). *Financial Regulatory Developments Focus*. *39*. https://www.shearman.com/-/media/Files/Perspectives/2018/10/Financial-Regulatory-Developments-Focus--Issue-39-October-5-2018-FIAFR-10052018.pdf?la=en&hash=9FFA4488F1F5BECB70A01332BA0C2FEA9310BCDA

[CR138] Sureshbhai PN, Bhattacharya P, Tanwar S (2020). KaRuNa: A blockchain-based sentiment analysis framework for fraud cryptocurrency schemes. IEEE International Conference on Communications Workshops (ICC Workshops).

[CR140] Torres, C. F., Steichen, M., & State, R. (2019). *The art of the scam: demystifying honeypots in ethereum smart contracts*.

[CR141] Toyoda, K., Ohtsuki, T., & Mathiopoulos, P. T. (2017). *Identification of high yielding investment programs in bitcoin via transactions pattern analysis*. *2018-Janua*, pp. 1–6. 10.1109/GLOCOM.2017.8254420

[CR142] Toyoda, K., Ohtsuki, T., & Mathiopoulos, P. T. (2018). *Multi-class bitcoin-enabled service identification based on transaction history summarization*. *September*, pp. 1129–1136. 10.1109/Cybermatics

[CR143] Toyoda, K., Ohtsuki, T., & Mathiopoulos, P. T. (2019). Time series analysis for bitcoin transactions: The case of Pirate@40’s HYIP scheme. *IEEE International Conference on Data Mining Workshops, ICDMW*, *2018-Novem*, pp. 151–155. 10.1109/ICDMW.2018.00028

[CR144] Toyoda K, Mathiopoulos PT, Ohtsuki T (2019). A novel methodology for HYIP operators’ bitcoin addresses identification. IEEE Access.

[CR146] U.S. Securities and Exchange Commission. (n.d.). *Insider Trading*. Investor.Gov. Retrieved 4 March 2021, from https://www.investor.gov/introduction-investing/investing-basics/glossary/insider-trading

[CR147] urRehman MH, Salah K, Damiani E, Svetinovic D (2020). Trust in blockchain cryptocurrency ecosystem. IEEE Transactions on Engineering Management.

[CR148] Vasek, M., Bonneau, J., Castellucci, R., Keith, C., & Moore, T. (2017). The Bitcoin Brain Drain: Examining the Use and Abuse of Bitcoin Brain Wallets. In J. Grossklags & B. Preneel (Eds.), *Financial cryptography and data security* (Vol. 9603, pp. 609–618). Springer Berlin Heidelberg. 10.1007/978-3-662-54970-4_36

[CR149] Vasek, M. (2017). *Measuring Bitcoin-based cybercrime*. University of Tulsa.

[CR150] Vasek, M., & Moore, T. (2018). *Analyzing the Bitcoin Ponzi scheme ecosystem*. 10.1007/978-3-662-58820-8_8

[CR151] Vasek M, Moore T, Böhme R, Okamoto T (2015). There’s no free lunch, even using Bitcoin: Tracking the popularity and profits of virtual currency scams. Financial cryptography and data security.

[CR152] Vrazel N (2019). Betting it all on the flip of a coin: Regulating cryptocurrency initial coin offerings and protecting investors. S. Tex. l. Rev..

[CR153] Waxenbaum S (2019). The SEC and ICOs: Connections between digital assets and citrus groves. SSRN Electronic Journal.

[CR154] Weber, K., Schütz, A. E., Fertig, T., & Müller, N. H. (2020). Exploiting the Human Factor: Social Engineering Attacks on Cryptocurrency Users. In P. Zaphiris & A. Ioannou (Eds.), *Learning and Collaboration Technologies. Human and Technology Ecosystems* (pp. 650–668). Springer International Publishing. 10.1007/978-3-030-50506-6_45

[CR155] Webroot. (2018). *Webroot Threat Research Review* [White Paper]. https://www-cdn.webroot.com/2415/4662/3914/Threat-Roundup-Review-Whitepaper.pdf

[CR156] Wright, J. (2018). *Don’t @ Me: Hunting Twitter Bots at Scale* (p. 43). Duo Security.

[CR157] Wu, J., Yuan, Q., Lin, D., You, W., Chen, W., Chen, C., & Zheng, Z. (2020). Who are the phishers? Phishing scam detection on ethereum via network embedding. *IEEE Transactions on Systems, Man, and Cybernetics: Systems*.

[CR158] Xia, P., Wang, H., Luo, X., Wu, L., Zhou, Y., Bai, G., Xu, G., Huang, G., & Liu, X. (2020). Don’t fish in troubled waters! characterizing coronavirus-themed cryptocurrency scams. *ArXiv Preprint * ArXiv:2007.13639.

[CR159] Xia P, Wang H, Zhang B, Ji R, Gao B, Wu L, Luo X, Xu G (2020). Characterizing cryptocurrency exchange scams. Computers & Security.

[CR160] Xie, R. (2019). *Why China had to “Ban” Cryptocurrency but the U.S. did not: A Comparative Analysis of Regulations on Crypto-Markets Between the U.S. and China*.

[CR161] Yli-Huumo J, Ko D, Choi S, Park S, Smolander K (2016). Where is current research on Blockchain technology?—A systematic review. PLoS ONE.

[CR162] Yuan Q, Huang B, Zhang J, Wu J, Zhang H, Zhang X (2020). Detecting Phishing scams on Ethereum based on transaction records. IEEE International Symposium on Circuits and Systems (ISCAS).

[CR163] Zetzsche, D., Buckley, R., & Amer, D. (2017). The ICO Gold Rush: It’s a scam, it’s a bubble, it’s a super challenge for regulators. In *EBI Working Paper Series* (Issue 18).

